# Computationally Empowered Workflow Identifies Novel Covalent Allosteric Binders for KRAS^G12C^

**DOI:** 10.1002/cmdc.201900727

**Published:** 2020-04-01

**Authors:** Jérémie Mortier, Anders Friberg, Volker Badock, Dieter Moosmayer, Jens Schroeder, Patrick Steigemann, Franziska Siegel, Stefan Gradl, Marcus Bauser, Roman C. Hillig, Hans Briem, Knut Eis, Benjamin Bader, Duy Nguyen, Clara D. Christ

**Affiliations:** ^1^ Bayer AG Research & Development, Pharmaceuticals Müllerstrasse 178 13342 Berlin Germany

**Keywords:** free-energy perturbation (FEP), KRAS, medicinal chemistry, molecular dynamics, virtual library design

## Abstract

Due to its frequent mutations in multiple lethal cancers, KRAS is one of the most‐studied anticancer targets nowadays. Since the discovery of the druggable allosteric binding site containing a G12C mutation, KRAS^G12C^ has been the focus of attention in oncology research. We report here a computationally driven approach aimed at identifying novel and selective KRAS^G12C^ covalent inhibitors. The workflow involved initial enumeration of virtual molecules tailored for the KRAS allosteric binding site. Tools such as pharmacophore modeling, docking, and free‐energy perturbations were deployed to prioritize the compounds with the best profiles. The synthesized naphthyridinone scaffold showed the ability to react with G12C and inhibit KRAS^G12C^. Analogues were prepared to establish structure‐activity relationships, while molecular dynamics simulations and crystallization of the inhibitor‐KRAS^G12C^ complex highlighted an unprecedented binding mode.

## Graphical abstract

**Discovery of a novel covalent KRAS^G12C^ inhibitor** with an unprecedented mode of binding in the switch‐II allosteric pocket. This is the outcome of a computationally driven workflow based on the enumeration of novel chemical matter and stepwise prioritization. The most promising compounds were synthesized, biochemically tested, and co‐crystallized with KRAS^G12C^.

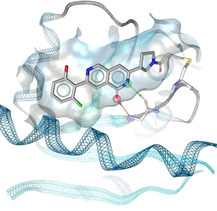

First identified in Kirsten rat sarcoma (KRAS) virus,^1^ mutations in the KRAS gene have widespread prevalence in cancers.^2^ In 1982, abnormally activated RAS genes were found in human cancers, marking the first discovery of mutated genes in this disease.^3^ The frequent mutation of RAS in three of the four most lethal cancers (lung, colon, and pancreatic cancers) in the United States has spurred intense interest and effort in developing RAS inhibitors.^4^ Overall, RAS mutations have been detected in 9–30 % of all tumor samples sequenced, with the specific RAS isoform generally differing according to cancer type. In pancreatic ductal adenocarcinoma and lung adenocarcinoma, there is a KRAS mutation frequency of 98 % and 31 %, respectively. In colon and rectal carcinoma (CRC), KRAS is also found predominantly in a mutated isoform (45 %), whereas NRAS mutations are infrequent (in 7.5 % of CRC) and HRAS mutations have not been detected.^4^ RAS proteins act as molecular switches alternating between an active, GTP‐bound state and an inactive, GDP‐bound state. Activated by guanine nucleotide exchange factors (GEFs), RAS in its GTP‐bound state interacts with a number of effectors.^5^ The return to the inactive state is driven by GTPase‐activating proteins (GAPs), which down‐regulate active RAS by accelerating the weak intrinsic GTPase activity by up to five orders of magnitude.^6^ For oncogenic RAS mutants, however, the GAP activity is impaired or greatly reduced, resulting in permanent activation, which is the basis of oncogenic RAS signaling.^7^

Mutation G12C in KRAS was recently identified to be potentially druggable by allele‐specific covalent inhibitors targeting the Cys12 side chain in vicinity to an inducible allosteric pocket, called the switch‐II pocket (also known as pocket 2).^8^ Occupation of this pocket with a covalently bound inhibitor results in a protein locked in an inactive GDP‐bound state. Locked in this conformation, the mutated KRAS cannot return to an active GTP‐bound state and activity of the G12C mutant is thereby shut down. Starting from Shokat's groundbreaking work on covalent KRAS^G12C^ inhibitors,8a Araxes progressed with various lead series, disclosing their strategy for the optimization of covalent KRAS^G12C^ inhibitors. Compound ARS‐853^9^ can be regarded as an in vitro tool compound, whereas quinazoline derivative ARS‐1620 has been used as an in vivo chemical probe to investigate KRAS^G12C^ biology.8b The first Araxes patent containing ARS‐1620 was published in 2015,^10^ and all inhibitors reported since then consist of minor structural variations of ARS‐1620 (Figure 1A). To the best of our knowledge, three companies have announced clinical trials so far: i) Amgen with AMG‐510 in 2018,^11^ ii) Mirati Therapeutics with MRTX849 in 2019,^12^ and iii) Araxes Pharma with ARS‐3248.^13^

**Figure 1:**
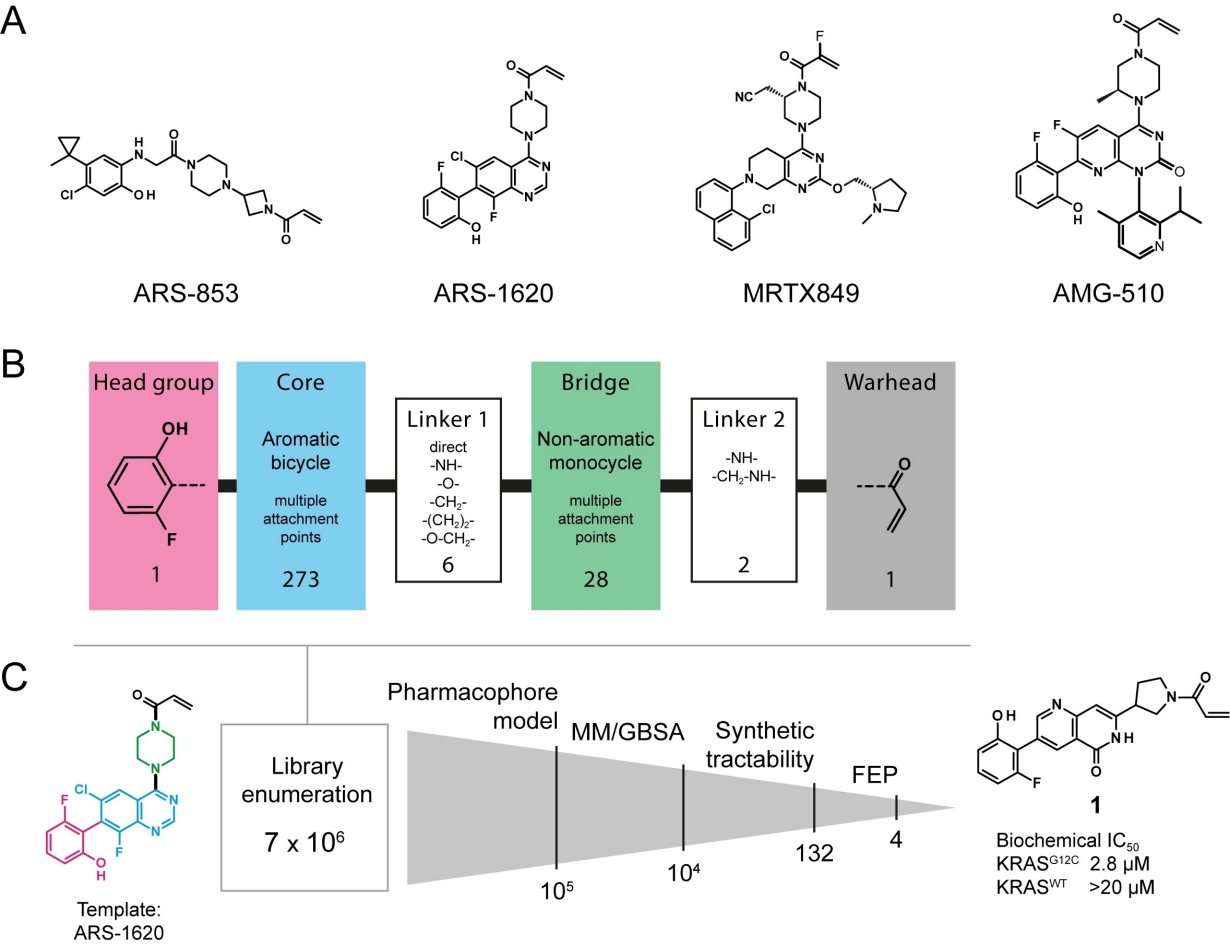
A) Inhibitors known to bind to the switch‐II pocket of KRAS^G12C^. B) Fragmentation of the inhibitor structure before enumeration, including a depiction of the nature and size of the used fragment libraries. C) Enumeration and prioritization workflow.

We report here a computationally driven methodology developed to identify novel chemical matter able to modulate KRAS^G12C^ activity through allosteric binding. With a view to generate chemical novelty while conserving the binding mode and potency of ARS‐1620, we deconstructed the molecule into four fragments: i) the acryloyl *warhead*, ii) the *bridge* piperazine, iii) the quinazoline *core*, and iv) the fluorophenol *head group* (Figure 1B). A computer‐aided scaffold hopping workflow was developed for the core fragment and the bridge, while the reactive acrylamide warhead and the fluorophenol head group were conserved (see the Supporting Information). The generated library includes almost 7×10^6^ compounds consisting of all possible building block combinations. None of these 7 million was found in the ChEMBL database,^14^ thus indicating the high novelty of the generated chemical matter. Five exact matches were found in SureChEMBL,^15^ all from the Araxes patent.^10^

Although the ARS compound series had been patented^10^ when our project was initiated, a binding mode had not been reported. Hence, one compound later confirmed as ARS‐1620 was modeled into the switch‐II pocket of KRAS^G12C^ (PDB entry 4LV6)8a using Cys12 as an anchor point, and then using molecular dynamics (MD) for refinement. The resulting trajectories allowed for the identification of a favored binding mode from which key interactions were extracted and compiled in one pharmacophore model (Figure S1 in the Supporting Information). Using Phase,^16^ the 7×10^6^ compound library was screened and, from the compounds that matched all pharmacophore features, the 10^5^ with the best alignment were retained (Figure 1C). In subsequent covalent docking, 10^4^ compounds were prioritized using MM/GBSA scoring, which balances computational efficiency and accuracy.^17^ To discard structures with low synthetic accessibility, the nucleophilicity of the position on the core aromatic fragment covalently bound to the bridge fragment was evaluated by visual inspection and compounds substituted at a position with poor electrophilicity were filtered out. Then, to allow for a rapid synthesis of the de novo designed compounds, the commercial availability of the required building blocks was evaluated. Eventually, a set of 132 compounds with tractable synthetic chemistry was prioritized.

At this stage of the project, ARS‐1620 had been successfully synthesized and co‐crystallized with KRAS^G12C^ in‐house, confirming the binding mode hypothesis previously used to generate the pharmacophore model (Figure S1). This allowed us to progress with the previously prioritized 132 compounds, and binding affinity estimates were calculated using free‐energy perturbations (FEP), a computationally expensive method that takes into account protein flexibility.^18^ Four compounds with calculated relative Δ*G* in the range to this of ARS‐1620 were prioritized.

One of the most synthetically accessible compounds, 1,6‐naphthyridin‐5(6*H*)‐one (**1**), was prepared (Table 1; Scheme S1). Structurally close to **1**, isoquinolin‐1(2*H*)‐one **2** derivatives were also considered for the exploration of structure‐activity relationships (SAR). To measure the proportion of inhibitor reacting with Cys12 of the KRAS^G12C^ mutant, the bound/unbound protein ratio was quantified by mass spectrometry (MS). Then, the inhibition potency of these compounds towards KRAS^G12C^ was evaluated in biochemical assays measuring the activation of GDP‐bound KRAS^G12C^ or KRAS^WT^ by SOS1.^19^

**Table 1:** Compound structures, KRAS^G12C^ binding measured by mass spectrometry (MS), and biochemical IC_50_ measurements.

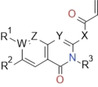										
Cmpd	R^1^	R^2^	R^3^	W	X	Y	Z	MS assay [% binding]	IC_50_ KRAS^G12C^ [μM]	IC_50_ KRAS^WT^ [μM]
**1**	H	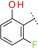	H	C	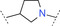	CH	N	80	2.8	>20
**2**	H	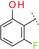	H	C	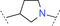	CH	CH	35	4.7	>20
**3**	H	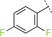	H	C	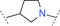	CH	CH	34	5.0	>20
**4**	H	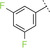	H	C	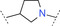	CH	CH	0	>20	>20
**5**	H	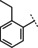	H	C	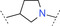	CH	CH	60	8.0	>20
**6**	H	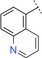	H	C	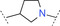	CH	CH	20	>16	>20
**7**	Cl	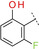	H	C	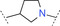	CH	CH	51	14.6	>20
**8**	H	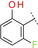	H	C	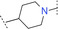	CH	CH	0	>20	>20
**9**	H	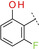	H	C	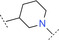	CH	CH	0	>20	>20
**10**	H	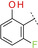	CH_3_	C	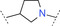	CH	CH	11	>20	>20
**11**	H	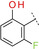	H	C	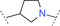	N	CH	59	4.90	>20
**12**	OH	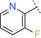	H	C	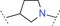	CH	CH	0	>20	>20
**13**	–	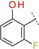	H	N	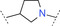	CH	CH	73	1.3	>20

Naphthyridinone **1** decorated with a fluorophenol head group and bridged to an acrylamide warhead through a pyrrolidine emerged as a promising novel KRAS^G12C^ inhibitor with a bound fraction of 80 % and IC_50_ value of 2.8 μM, while no inhibition of KRAS^WT^ was observed up to a concentration of 20 μM (Table 1). Quinazolinone **11** and isoquinolinone **2** (with one nitrogen atom less than **1**) displayed a KRAS^G12C^‐bound fraction of 59 % and 35 %, respectively. Their measured KRAS^G12C^ IC_50_ values were below 5 μM (4.9 μM and 4.7 μM, respectively). A series of boronic acids were coupled to the corresponding bromo‐substituted isoquinolinone to yield four analogues **3**–**6** (Table 1, also see the Supporting Information). While a complete loss of activity was observed when the fluorophenol moiety was replaced by a quinolinyl (**6**) or a difluorophenyl (**4**) group, conserving the *ortho*‐fluorine and replacing the hydroxy group by a *para*‐fluoro substituent (**3**) or an *ortho*‐ethyl group (**5**) allows for the inhibitor to modulate KRAS^G12C^ activity in the low micromolar range (5.0 μM and 8.0 μM, respectively). Also, alternatives to the pyrrolidine bridge were investigated, with the 4‐ and 3‐piperidinyl derivatives (**8** and **9**, respectively) both being inactive. The isoquinolinone variant **10** decorated with a methyl on the core nitrogen shows a similar inability to bind KRAS^G12C^, highlighting the importance of the hydrogen‐bond donor at this position.

Crystallization trials were initiated and a crystal structure of **3** in complex with KRAS^G12C^/GDP was determined at high resolution (2.0 Å). As suggested by MD (see the Supporting Information), the covalent inhibitor does exhibit a different binding mode compared to ARS‐1620. The most obvious difference is the rotation of the isoquinolinone core, which allows direct hydrogen bonding between the ligand and the backbone atoms of Gly10. In contrast, the carbonyl oxygen of the covalent warhead is coordinated by Lys16 in a very similar fashion as the respective moiety in ARS‐1620. Also, the terminal difluorophenyl head group is essentially in the same subpocket and orientation as that of the fluorophenol in ARS‐1620. Consequently, the isoquinolinone core and the head group are closer to a coplanar orientation (146 °), unlike ARS‐1620 (70 °), as illustrated in Figure 2A–F. In a retrospective analysis of the trajectories resulting from the FEP calculations, a similar rotation of the core fragment was detected for **1**, explaining why this scaffold was selected in the initial screening campaign (Figure S3). This result mirrors the SAR, demonstrating a novel mode of binding for this series.

**Figure 2:**
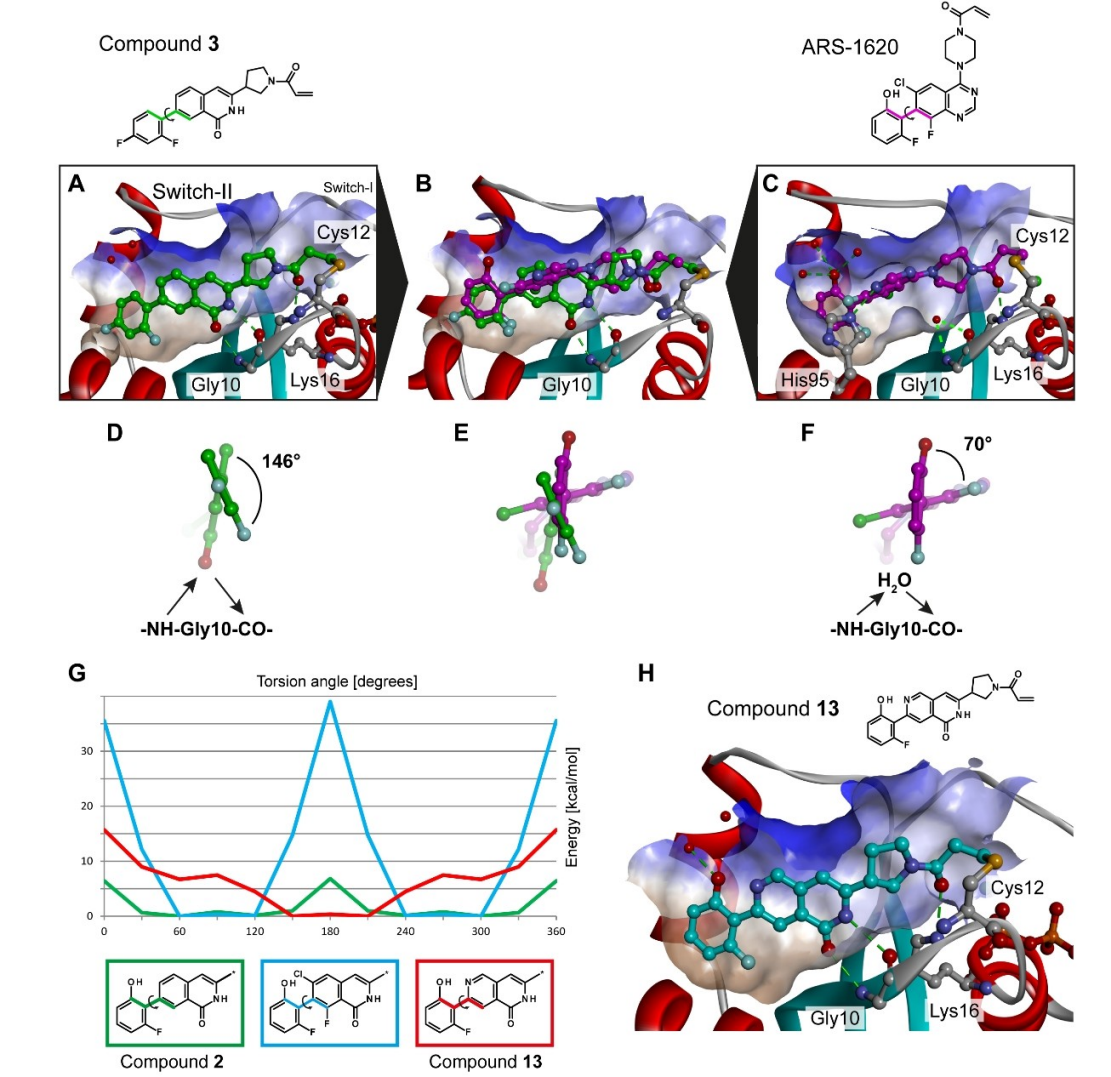
Co‐crystal structures of KRAS^G12C^ with compound **3** (A, PDB entry: 6TAM) and ARS‐1620 (C), and an overlay of both ligands (B). Extracting these particular conformations, the head group of compound **3** (D) is compared to that of ARS‐1620 (F), with a superimposition of both (E); in the case of **3**, the isoquinolinone core rotates, replacing the water molecule observed in the co‐crystal structure with ARS‐1620 and allowing a direct interaction with Gly10. G) Comparison of the energy landscape of the dihedral of fragments from compounds **2**, **13**, and a virtual analogue with Cl and F atoms flanking the isoquinolinone core (* indicates the position of the compound fragmentation for the QM study). H) Co‐crystal structure of KRAS^G12C^ with compound **13** (PDB entry: 6TAN).

In the isoquinolinone series, quantum chemical calculations suggested that adding decorating groups at the ortho positions of the phenyl moiety energetically disfavors a coplanar orientation (Figure 2G). Similarly, adding a chloro substituent to the isoquinolinone core (**7**) was suggested to have a negative effect on the binding affinity. However, introducing one nitrogen atom at the same position of the core scaffold should allow for the formation of an intramolecular hydrogen bond with the fluorophenol head group. The energy landscape of the dihedral between the 2,6‐naphthyridin‐1(2*H*)‐one core (**13**) and the fluorophenol head group indicates that an energy minimum is reached at a dihedral angle between 150 ° and 210 ° (Figure 2G), the range of the active conformation. As these results highlight the potential of stabilizing a coplanar orientation with an intramolecular hydrogen bond, **12** and **13** were synthesized and tested.

The MS covalent binding assay revealed that **12** is unable to react with Cys12 of KRAS^G12C^ (Table 1); this, indicates that the hydroxy group flanking the isoquinolinone core might be inadequate for optimal binding. However, inversing the intramolecular hydrogen bond allows for the fluorophenol moiety to be conserved: 2,6‐naphthyridinone **13** reacts with Cys12 of KRAS^G12C^, with a bound fraction of 73 %, and shows a slightly improved IC_50_ value (1.3 μM) compared to the analogue with no intramolecular hydrogen bond (**2**, IC_50_=4.7 μM). The binding constant of the reversible binding event (*K*_I_) and the maximum potential rate of inactivation (*k*_inact_) were measured for compounds **1**, **2** and **13**. These results indicate a weak *k*_inact_,^20^ and a *k*_inact_/*K*_I_ ratio in the same range for the three compounds (Table 2). Finally, cellular activity was detected for compound **13** (IC_50_=22 μM) and the crystal structure of KRAS^G12C^ in complex with **13** confirmed its binding mode (Figure 2H). These results provide the foundation for further optimization, aiming for sub‐micromolar cellular activity.

**Table 2:** Measurements of binding constant of the reversible binding event (K_I_), maximum potential rate of KRAS^G12C^ inactivation (k_inact_), and cellular activity.

Cmpd	*K*_I_ [μM]	*k*_inact_ [1/s]	*k*_inact_/*K*_I_ [1/(M*s)]	Cellular IC_50_ [μM]
**1**	39.3	0.00105	26.72	>30
**2**	4.4	0.00024	55.05	>30
**13**	32.2	0.00159	49.38	22

Interestingly, free‐binding‐energy calculations using FEP+^21^ could not accurately anticipate the effect of this intramolecular hydrogen bond. The relative Δ*G* of **12** and **13** was greatly overestimated, predicting an improvement of about 100‐fold in binding affinity compared to **2**. On the other hand, the involvement of protein dynamics and free energy calculations in our workflow was key to the identification of a scaffold with a binding mode unprecedented since the discovery of the KRAS^G12C^ allosteric pocket. With the presented computer‐aided approach coupled with a stepwise experimental validation, we have reported here the design of a novel chemical series binding to KRAS^G12C^ with high potential for the development of pioneering KRAS‐targeted anti‐cancer treatments.

## Conflict of interest

All authors are current or former employees of Bayer AG.

## Supporting information

As a service to our authors and readers, this journal provides supporting information supplied by the authors. Such materials are peer reviewed and may be re‐organized for online delivery, but are not copy‐edited or typeset. Technical support issues arising from supporting information (other than missing files) should be addressed to the authors.

Supplementary
