# Supplementary material for: Computationally Empowered Workflow Identifies Novel Covalent Allosteric Binders for KRASG12C
Source: ChemMedChem. 2020 Apr 1;15(10):827–32. doi: 10.1002/cmdc.201900727 (PMC7318243; doi:10.1002/cmdc.201900727)
Supplement: Supplementary file 1 — Supplementary [file CMDC-15-827-s001.pdf]

ChemMedChem

Supporting Information

Computationally Empowered Workflow Identifies Novel Covalent Allosteric Binders for $KRAS^{G12C}$

Jérémie Mortier,* Anders Friberg, Volker Badock, Dieter Moosmayer, Jens Schroeder, Patrick Steigemann, Franziska Siegel, Stefan Gradl, Marcus Bauser, Roman C. Hillig, Hans Briem, Knut Eis, Benjamin Bader, Duy Nguyen,* and Clara D. Christ*© 2020 The Authors. Published by Wiley-VCH Verlag GmbH & Co. KGaA.
This is an open access article under the terms of the Creative Commons Attribution License, which permits use, distribution and reproduction in any medium, provided the original work is properly cited.

Author Contributions

A.F. Conceptualization:Lead; Investigation:Lead; Writing - Original Draft:Supporting; Writing - Review & Editing:-Supporting

SUPPORTING INFORMATION

Table of Contents

1. Computational methods S3
1.1 Initial model for ARS-1620 binding S3
1.2 Switch-II-focused virtual library of ligands S4
1.3 Virtual library screening S4
1.4 Molecular dynamics simulations S5
1.5 Quantum mechanics S6
2. Chemistry S7
2.1 General synthetic strategy S7
2.2 Methods and materials S8
2.3 Compound syntheses S8
2.3.1 3-(2-Fluoro-6-hydroxyphenyl)-7-[1-(prop-2-enoyl)pyrrolidin-3-yl]-1,6-naphthyridin-5(6*H*)-one (**1**) S8
2.3.2 7-(2-Fluoro-6-hydroxyphenyl)-3-[1-(prop-2-enoyl)pyrrolidin-3-yl]isoquinolin-1(2*H*)-one (**2**) S9
2.3.3 7-(2,4-Difluorophenyl)-3-[1-(prop-2-enoyl)pyrrolidin-3-yl]isoquinolin-1(2*H*)-one (**3**) S11
2.3.4 7-(3,5-Difluorophenyl)-3-[1-(prop-2-enoyl)pyrrolidin-3-yl]isoquinolin-1(2*H*)-one (**4**) S11
2.3.5 7-(2-Ethylphenyl)-3-[1-(prop-2-enoyl)pyrrolidin-3-yl]isoquinolin-1(2*H*)-one (**5**) S11
2.3.6 3-[1-(Prop-2-enoyl)pyrrolidin-3-yl]-7-(quinolin-5-yl)isoquinolin-1(2*H*)-one (**6**) S12
2.3.7 6-Chloro-7-(2-fluoro-6-hydroxyphenyl)-3-[1-(prop-2-enoyl)pyrrolidin-3-yl]isoquinolin-1(2*H*)-one (**7**) S12
2.3.8 6-(2-Fluoro-6-hydroxyphenyl)-2-[1-(prop-2-enoyl)pyrrolidin-3-yl]quinazolin-4(3*H*)-one (**11**) S13
2.3.9 7-(2-Fluoro-6-hydroxyphenyl)-3-[1-(prop-2-enoyl)pyrrolidin-3-yl]-2,6-naphthyridin-1(2*H*)-one (**13**) S14
3. Protein production, crystallization, and mass spectrometry S17
3.1 Protein preparation S17
3.2 Crystallography S17
3.2.1 Compound **3** S17
3.2.2 Compound **13** S17
3.2.3 Data collection and refinement S17
3.3 Biochemical activity assays S18
3.4 Covalent binding assay by mass spectrometry S19
3.5 Cellular assay for RAS activation S19
3.6 Determination of k_{inact} and K_I S19
4. References S21

1. Computational methods

1.1 Initial model for ARS-1620 binding

The initial model of KRAS^{G12C} bound to ARS-1620 was built using entry 4LV6 retrieved from the Protein Data Bank (PDB).[1] The structure was prepared with Maestro[2] using the Protein Preparation Wizard[3] as follows: All water molecules were retained. Prime was used to fill missing loops and side chains, and the GDP nucleotide was conserved in the KRAS active site.[4] Side-chain protonation states were assigned at pH 7, after which the hydrogen-bonding network was optimized using default parameters. Once the original ligand was extracted, a rough binding mode resulting from a nucleophilic attack of the Cys12 side chain was hypothesized by positioning compound ARS-1620 into the binding pocket and removing clashing water molecules. Then, a molecular dynamics (MD) simulation was conducted using Desmond.[5] The system was solvated in an orthorhombic box of SPC water[6] using a buffer distance of 10 Å, the system was neutralized, and sodium and chlorine ions were added to obtain a salt concentration of 0.15 M. The OPLS3 force field was applied.[7] The system was relaxed using the default protocol and then simulated for 50 ns at 300 K using a Langevin thermostat and barostat[8] and default parameters for all other settings. The trajectory was visually inspected, and one frame considered to be a good representation of the interaction network was selected as binding mode model. This frame was extracted from the trajectory and a pharmacophore model was derived from this structure using Phase.[9] The pharmacophore model contains six features (Figure S1) and exclusion volumes: one aromatic feature (matching tolerance 2 Å) and one hydrophobic feature (1 Å) on the fluorophenol head group in order to correctly position this conserved moiety; two aromatic features on the core (2 Å) as well as one acceptor feature on the nitrogen at the 3-position (1 Å). The sixth feature was selected to correctly position the carbonyl oxygen acceptor (1 Å). This pharmacophore model was used to filter the virtual library of ~7 × 10^6 compounds.

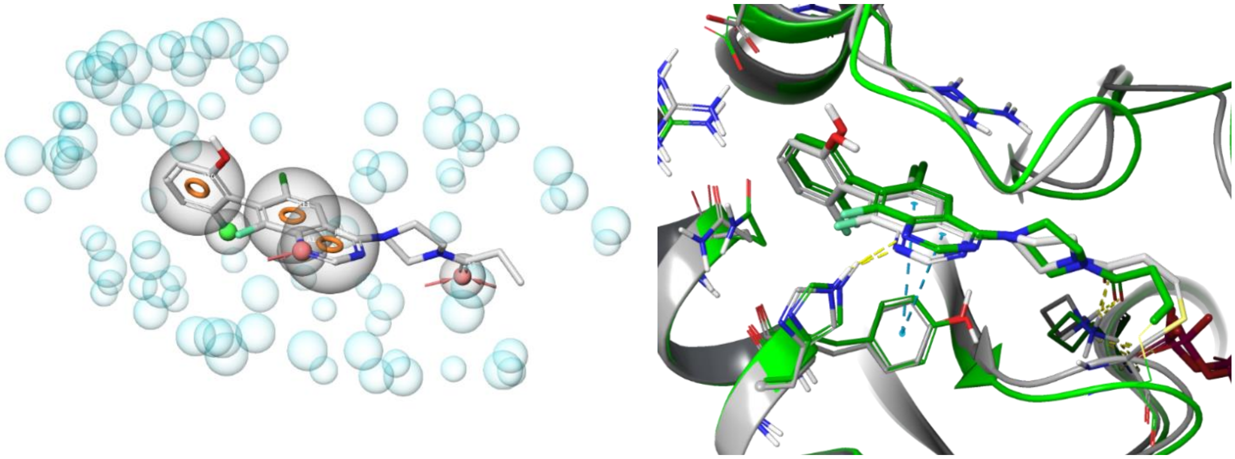

Figure S1. Left: Pharmacophore model developed from the ARS-1620 binding hypothesis and used for virtual screening. Right: Crystal structure of the KRAS^{G12C}–ARS-1620 complex (green) superimposed on the binding hypothesis (white) built using entry 4LV6 retrieved from the Protein Data Bank.[1]

The binding mode hypothesis used to generate the pharmacophore model was confirmed later with a co-crystal structure of KRAS^{G12C} bound to ARS-1620 (Figure S1), also in good agreement with the structure published subsequently (PDB entry 5V9U).[10]

1.2 Switch-II-focused virtual library of ligands

A library of ligands structurally similar to ARS-1620 and likely to bind to the Switch-II allosteric site of KRAS was enumerated computationally by combining a *head group*, a *core*, a *bridge*, and a *warhead*. The only *head group* considered in this enumeration was the fluorophenol moiety and the only *warhead*, an acrylamide. Using VEHICLe,[11] 273 aromatic bicycles with high precedence in the literature (>1000 occurrences) were collected. To substitute the piperazine *bridge*, 28 non-aromatic monocycles were assembled manually (Figure S2). Then, a virtual library was constructed by systematically enumerating all possible combinations of one *head group* (fluorophenol moiety) + one *core* (273 scaffolds) + one *bridge* (28 scaffolds) + one *warhead* (Michael acceptor acrylamide), allowing multiple attachment points between each fragment. Linking of the *head group* and the *bridge* was allowed on all C atoms of the *core* scaffold, except those atoms belonging to both rings of the bicyclic ring system (i.e., bridgeheads). The *head group* was always attached directly to a *core* carbon. The *bridge* was covalently linked to the *core* directly or with one of the following bridging groups: (i) -O-, (ii) -NH-, (iii) -CH_2-, (iv) -CH_2-CH_2-, or (v) -CH_2-O-. Acrylamide was the *warhead* systematically used to cap all enumerated chemical structures. The *bridge* fragment was then attached directly to the acryloyl portion of the *warhead* (i) by a ring nitrogen already present in the *bridge*, (ii) by an additional -NH-, or (iii) by an additional -CH_2-NH-. The generated virtual library including all possible combinations of these building blocks contained 6,955,092 virtual compounds.

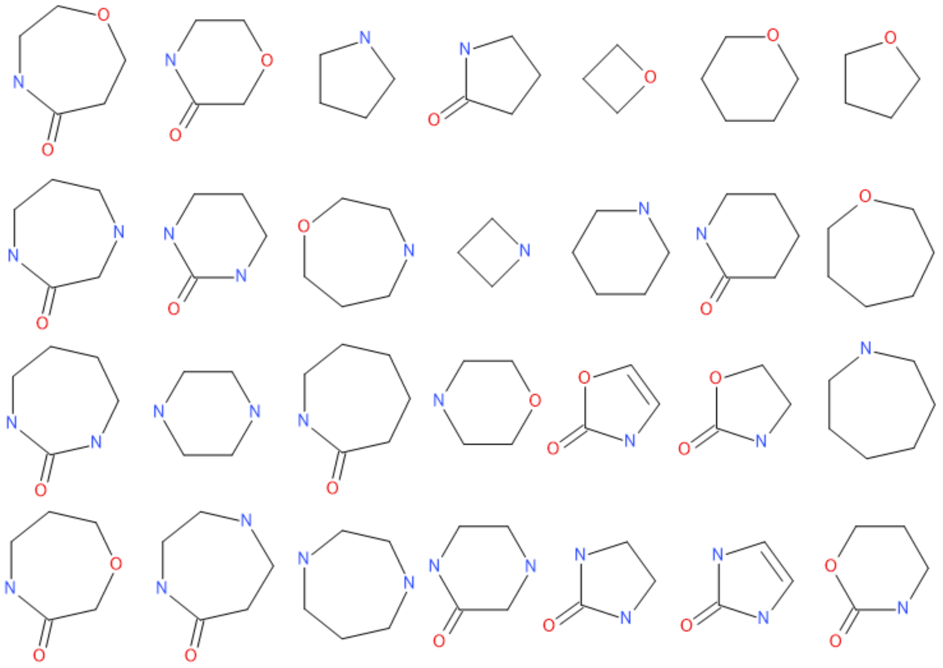

Figure S2. Structures of the 28 non-aromatic monocyclic bridges used in the library enumeration.

1.3 Virtual library screening

A maximum of 50 conformations were generated in three dimensions on-the-fly for each virtual ligand using Phase in rapid mode.[9] From the compounds that matched all hypothesis sites, the 10^5 compounds with the best alignment to the pharmacophore model were selected for covalent docking. During covalent docking,[2, 12] a Michael addition of the acrylamide warhead to cysteine 12 was carried out. As the pharmacophore search positioned the virtual molecules already inside

the binding cavity, covalent docking was performed in an approximate fashion using only minimization in place as sampling method within the Glide sub-step. MM/GBSA[13] scoring was performed and the top scoring 10000 molecules were retained for further prioritization.
An in-house crystal structure of ARS-1620 (data not shown; essentially identical to later PDB deposition by Janes et al.: 5V9U)[10] was prepared as described previously (see details above for 4LV6, Section 1.1). Free-binding energy calculations were carried out in a first round of FEP with the 132 compounds considered synthetically tractable. As the covalent scaffold hopping FEP+ module was not available at the time, molecules covalently linked were considered together with the cysteine side chain and their free-binding energy was calculated relatively to a free glycine side chain, used as a reference (1 ns/edge). This approach was necessary as minor difference in cysteine 12 conformations led to non-optimally overlapping ligand starting conformations. A total of 20 compounds chosen from the set of 132 molecules with promising and well-converged free-binding energies were selected for a second round of covalent FEP calculations (10 ns/edge). Apart from the simulation time, default FEP+ parameters were used in Maestro.[2, 14]

1.4 Molecular dynamics simulations

To further investigate the binding mode of the isoquinolinone scaffold in the allosteric binding site of KRAS, a (non-covalent) docking study was conducted with compound **2** using Glide[15] in Maestro.[2] Default parameters and zero constraints were used, defining the binding pocket of $KRAS^{G12C}$ by a volume of 6 Å around the co-crystallized ligand ARS-1620. The generated binding modes are non-covalent or, in other words, before the reaction with the side chain of cysteine 12. The closest docking conformation to this of the co-crystallized ARS-1620 was selected as starting conformation to conduct MD simulations with explicit water molecules using Desmond.[5] Using the OPLS3 force field,[7] the $KRAS^{G12C}$–ARS-1620 complex was placed in a cubic box filled with explicit water molecules using a flexible simple point-charge (SPC) model and salt (0.15 M NaCl). Minimization and equilibration were performed using default parameters and the enzyme–ligand complex was simulated for 50 ns with periodic boundary conditions. A pressure of 1.01325 bars was kept constant using the Martyna–Tobias–Klein barostat method[16] and a temperature of 300 K was kept constant with a Nose–Hoover thermostat.[17] Short-range electrostatic interactions were calculated up to a 9 Å distance cutoff. With RESPA integrator, constraints were used on all bonds with 2 fs time step length for bonded and near atoms, and 6 fs for far atoms.
Trajectory analysis indicated that the isoquinolin-1(2*H*)-one fragment rotates about 90° until its NH-CO moiety is oriented towards Gly10, forming a hydrogen-bond network with its backbone CO and NH groups (Figure 2A). In $KRAS^{G12C}$ co-crystallized with ARS-1620, a water molecule is detected between the inhibitor and Gly10, interacting with its backbone NH (Figure 2C). In an MD simulation, the binding mode of ARS-1620 and the position of the water molecule remained unchanged. Contrarily, the same water molecule conserved for the docking and present in the starting conformation was ejected and replaced by compound **2**. After a retrospective analysis of the trajectories resulting from the FEP calculations, a similar rotation of the core fragment was detected for compound **1**, explaining why this scaffold was selected in the initial screening campaign. This result mirrors the SAR (**10**, the *N*-Me variant of **2**, is inactive), indicating a novel mode of binding for this series (Figure S3). This binding mode was confirmed eventually by crystallography (see Section 3.2).

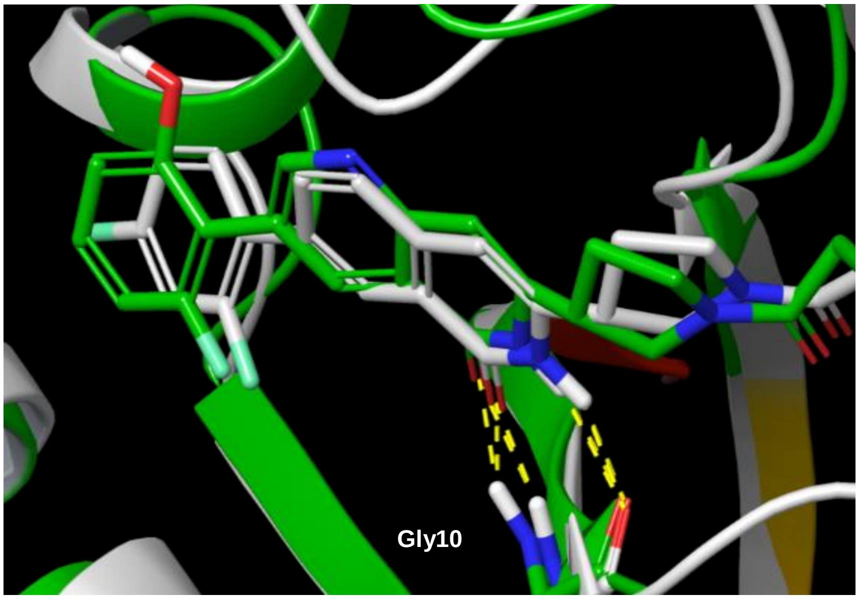

Figure S3. Comparison of last FEP frame of **1** (green) to the crystal structure (gray) of **3**, with hydrogen bonds highlighted in yellow.

1.5 Quantum mechanics

The 3D conformation energy landscape analysis was performed using Forcefield Builder,[7,18] implemented in Maestro using default parameters.

2. Chemistry

2.1 General synthetic strategy

The synthesis of compounds **1** and **2** is outlined in Scheme S1. Starting from 5-bromo-2-iodopyridine-3-carbonitrile (**14**), compound **15** was obtained by Sonogashira coupling with *tert*-butyl 3-ethynylpyrrolidine-1-carboxylate. Subsequent conversion of the cyano group into the corresponding carboxamide **16** was followed by cyclization to afford 1,6-naphthyridin-5(6*H*)-one **17**. Removal of the protecting group and subsequent introduction of the warhead by reaction with prop-2-enoyl chloride delivered **18**, which underwent Suzuki coupling with (2-fluoro-6-hydroxyphenyl)boronic acid to deliver compound **1**.

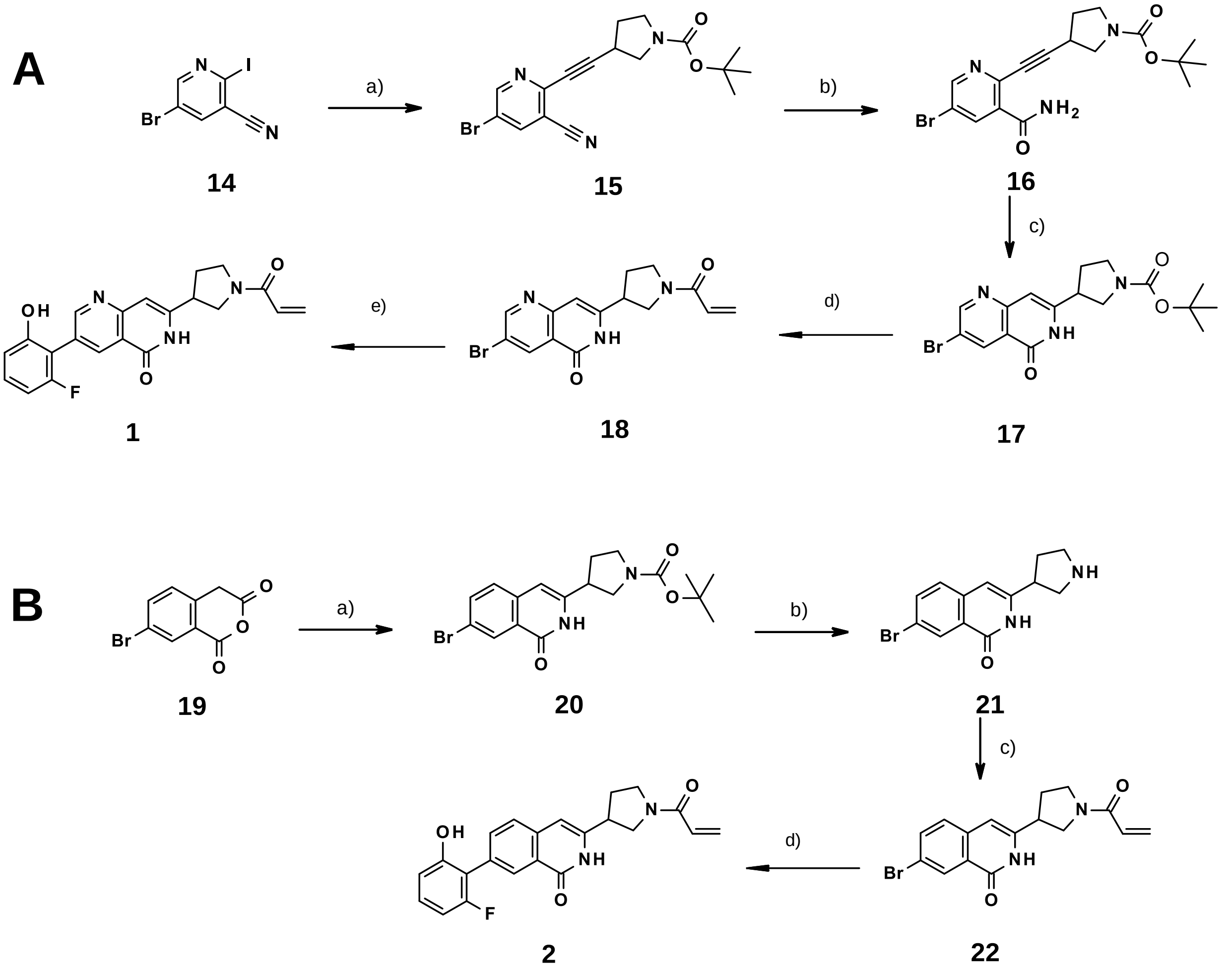

Scheme S1. A) Synthesis of 1,6-naphthyridin-5(6*H*)-one **1**. *Reagents and conditions*: (a) *tert*-butyl 3-ethynylpyrrolidine-1-carboxylate, triethylamine, CuI, $Pd(PPh_3)_4$, toluene, 70 °C, 24 h (52%); (b) NaOH, H_2O_2, MeOH, 0 °C, 2 h (52%); (c) NaH, THF, 10 °C; then RT, 5 h (30%); (d) TFA, DCM, RT, 2 h; then prop-2-enoyl chloride, (*i*-Pr)$_2$NEt, DCM, RT, overnight (90% over 2 steps); (e) (2-fluoro-6-hydroxyphenyl)boronic acid, RuPhos/RuPhos Pd G3, K_2CO_3, 1,4-dioxane/water (3:1), 65 °C, 18 h (18%). B) Synthesis of isoquinolin-1(2*H*)-one **2**. *Reagents and conditions*: (a) *tert*-butyl 3-(chlorocarbonyl)pyrrolidine-1-carboxylate, pyridine, RT; aqueous NH_4OH, reflux, 6 h; (b) HCl, THF, RT, 2 h; (c) prop-2-enoyl chloride, (*i*-Pr)$_2$NEt, DCM, RT, overnight; (d) (2-fluoro-6-hydroxyphenyl)boronic acid, RuPhos/RuPhos Pd G3, K_2CO_3, 1,4-dioxane/water (3:1), 65 °C, 18 h (15%).

Isoquinolin-1(2*H*)-one **2** was synthesized starting from commercially available 7-bromo-1*H*-isochromene-1,3(4*H*)-dione (**19**). Acylation of **19** was carried out using *tert*-butyl 3-

(chlorocarbonyl)pyrrolidine-1-carboxylate and subsequent treatment with ammonium hydroxide resulted in the formation of compound **20**. Cleavage of the protecting group yielded the intermediate compound **21**, which was then converted into compound **22** by amide coupling with prop-2-enoyl chloride. Suzuki coupling with (2-fluoro-6-hydroxyphenyl)boronic acid delivered isoquinolinone **2** in 7% overall yield.

2.2 Methods and materials

Proton NMR spectra were recorded using a Bruker Avance III HD 400 MHz or 500 MHz spectrometer. All deuterated solvents contained typically 0.03% to 0.05% v/v tetramethylsilane, which was used as the reference signal (set at δ 0.00 for ^{1}H). Measurements were performed at room temperature, unless otherwise stated.

LC-MS Method 1: instrument: Agilent 1290 UHPLC-MS Tof; column: BEH C18 (Waters) 1.7 µm, 50 × 2.1 mm; eluent A: water + 0.05 vol% formic acid (99%), eluent B: acetonitrile + 0.05 vol% formic acid (99%); gradient: 0–1.7 min 2–90% B, 1.7–2 min 90% B, 2–2.5 min 90–2% B; flow: 1.2 mL/min; temperature: 60 °C; DAD scan: 210–400 nm.

LC-MS Method 2: instrument: Waters Acquity UPLC-MS Single Quad; column: Acquity BEH C18 1.7 µm, 50 × 2.1 mm; eluent A: water + 0.1 vol% formic acid (99%), eluent B: acetonitrile; gradient: 0–1.6 min 1–99% B, 1.6–2.0 min 99% B; flow 0.8 mL/min; temperature: 60 °C; DAD scan: 210–400 nm.

HPLC Method 1: instrument: Waters AutoPurification system; column: Waters XBridge C18 5 µm, 100 × 30 mm; eluent A: water + 0.1 vol% formic acid (99%), eluent B: acetonitrile; DAD scan: 210-400 nm.

HPLC Method 2: instrument: Waters AutoPurification system; column: Waters XBridge C18 5 µm, 100 × 30 mm; eluent A: water + 0.2 vol% aqueous ammonia (32%), eluent B: acetonitrile; DAD scan: 210-400 nm.

2.3 Compound syntheses

2.3.1 3-(2-Fluoro-6-hydroxyphenyl)-7-[1-(prop-2-enoyl)pyrrolidin-3-yl]-1,6-naphthyridin-5(6*H*)-one (**1**) (see Scheme S1)

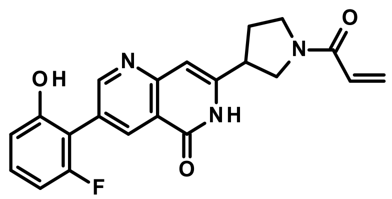

Step 1. 5-Bromo-2-iodopyridine-3-carbonitrile (**14**): A mixture of 5-bromo-2-chloropyridine-3-carbonitrile (40 g, 184 mmol), propionitrile (500 mL), and TMSI (50 g, 250 mmol) was stirred at reflux for 12 h. After evaporation to dryness, the crude was poured into water and extracted with MTBE. The combined organic layer was dried with Na_2SO_4 and concentrated under reduced pressure to give 51.6 g (167 mmol, 90% yield) of the title compound.

Step 2. *tert*-Butyl 3-[2-(5-bromo-3-cyano-2-pyridyl)ethynyl]pyrrolidine-1-carboxylate (**15**): To a solution of 5-bromo-2-iodopyridine-3-carbonitrile (**14**; 10 g, 32.4 mmol), *tert*-butyl 3-ethynyl-pyrrolidine-1-carboxylate (6.32 g, 32.4 mmol), and triethylamine (50 mL, 359 mmol) in toluene

(100 mL) under argon atmosphere were added CuI (0.124 g, 0.651 mmol) and $Pd(PPh_3)_4$ (0.75 g, 0.649 mmol). The mixture was stirred at 70 °C for 24 h. After the solvent was evaporated under reduced pressure, water (500 mL) was added to the residue which was followed by extraction with MTBE. The combined organic layers were dried with Na_2SO_4, filtered, and concentrated under reduced pressure. The crude product was purified by column chromatography to obtain 6.46 g (17.17 mmol, 52% yield) of the title compound.

Step 3. *tert*-Butyl 3-[2-(5-bromo-3-carbamoyl-2-pyridyl)ethynyl]pyrrolidine-1-carboxylate (**16**): To a solution of *tert*-butyl 3-[2-(5-bromo-3-cyano-2-pyridyl)ethynyl]pyrrolidine-1-carboxylate (**15**; 12.5 g, 33.2 mmol) and NaOH (1.2 g, 30.0 mmol) in MeOH (200 mL) was added, at 0 °C, 30% aqueous hydrogen peroxide (3.75 mL, 36.52 mmol). The reaction mixture was stirred for 2 h at 0 °C, then concentrated under reduced pressure. The residue was diluted with water, and the precipitate was collected by filtration and dried under reduced pressure to give 9.56 g (24.24 mmol, 73% yield) of the title compound.

Step 4. *tert*-Butyl 3-(3-bromo-5-oxo-5,6-dihydro-1,6-naphthyridin-7-yl)pyrrolidine-1-carboxylate (**17**): To a solution of *tert*-butyl 3-[2-(5-bromo-3-carbamoyl-2-pyridyl)ethynyl]pyrrolidine-1-carboxylate (**16**; 7 g, 17.8 mmol) in THF (100 mL) was added, in small portions, 60% NaH in mineral oil (1.5 g, 37.5 mmol) at 10 °C. After 5 h at room temperature, the reaction mixture was poured onto ice and then extracted with MTBE (3 × 250 mL). The combined organic layers were washed with 1 N citric acid solution, dried over Na_2SO_4, and concentrated under reduced pressure. The crude product was purified by column chromatography to obtain 2.11 g (5.34 mmol, 30% yield) of the title compound. ^{1}H NMR (500 MHz, [D$_6$]DMSO): δ [ppm] = 1.40 (s, 9H), 2.05–2.20 (m, 2H), 3.40–3.50 (m, 1H), 3.70–3.80 (m, 1H), 6.45 (s, 1H), 8.50 (s, 1H), 9.00 (s, 1H), 11.75 (s, 1H); some pyrrolidine resonances overlap with a water resonance.

Step 5. 3-Bromo-7-(pyrrolidin-3-yl)-1,6-naphthyridin-5(6*H*)-one: To a solution of *tert*-butyl 3-(3-bromo-5-oxo-5,6-dihydro-1,6-naphthyridin-7-yl)pyrrolidine-1-carboxylate (**17**; 1.97 g, 4.99 mmol) in THF (19 mL) was added 4 M HCl in 1,4-dioxane (71 mL, 285 mmol). After 2 h, the reaction mixture was concentrated under reduced pressure to give 1.47 g of the title compound which was used in the next step without prior characterization.

Step 6. 3-Bromo-7-[1-(prop-2-enoyl)pyrrolidin-3-yl]-1,6-naphthyridin-5(6*H*)-one (**18**): Prepared from 3-bromo-7-(pyrrolidin-3-yl)-1,6-naphthyridin-5(6*H*)-one (1.47 g) in an analogous fashion as described for compound **22** to give 1.57 g (90% yield over 2 steps) of the title compound as a mixture of rotamers. ^{1}H NMR (400 MHz, [D$_6$]DMSO): δ [ppm] = 2.02–2.38 (m, 4H), 3.37–3.52 (m, 4H), 3.57–3.80 (m, 4H), 3.92–3.94 (m, 1H), 4.00–4.08 (m, 1H), 5.62–5.73 (m, 2H), 6.11–6.21 (m, 2H), 6.49 (s, 1H), 6.55 (s, 1H), 6.57–6.66 (m, 2H), 8.46–8.63 (m, 2H), 8.94–9.01 (m, 2H), 11.83 (br s, 2H).

Step 7. 3-(2-Fluoro-6-hydroxyphenyl)-7-[1-(prop-2-enoyl)pyrrolidin-3-yl]-1,6-naphthyridin-5(6*H*)-one (**1**): Prepared from 3-bromo-7-[1-(prop-2-enoyl)pyrrolidin-3-yl]-1,6-naphthyridin-5(6*H*)-one (**18**; 100 mg, 0.287 mmol) in an analogous fashion as described for compound **2** to give 21 mg (18% yield) of the title compound. ^{1}H NMR (500 MHz, [D$_6$]DMSO, 80 °C): δ [ppm] = 2.07–2.41 (m, 2H), 3.28–4.20 (m, 5H), 5.68 (dd, *J*=10.33, 2.38 Hz, 1H), 6.16 (dd, *J*=16.85, 2.54 Hz, 1H), 6.49–6.66 (m, 2H), 6.76–6.81 (m, 1H), 6.87 (d, *J*=8.27 Hz, 1H), 7.23–7.29 (m, 1H), 8.44–8.47 (m, 1H), 8.67–8.88 (m, 1H), 9.93 (br s, 1H), 11.43 (br, 1H).

2.3.2 7-(2-Fluoro-6-hydroxyphenyl)-3-[1-(prop-2-enoyl)pyrrolidin-3-yl]isoquinolin-1(2*H*)-one (2) (see Scheme S1)

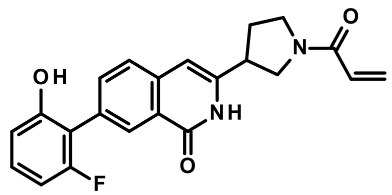

Step 1. *tert*-Butyl 3-(7-bromo-1-oxo-1,2-dihydroisoquinolin-3-yl)pyrrolidine-1-carboxylate (**20**): To a solution of commercially available 7-bromo-1*H*-isochromene-1,3(4*H*)-dione (**19**; 308 mg, 1.28 mmol) in pyridine (27 mL) was added *tert*-butyl 3-(chlorocarbonyl)pyrrolidine-1-carboxylate (299 mg, 1.28 mmol). The reaction mixture was stirred at room temperature overnight, then diluted with toluene. After removal of the volatiles, 25% aqueous NH_4OH (20 mL, 128 mmol) was added to the crude product (686 mg). The mixture was heated (reflux) for 6 h. Removal of the volatiles and subsequent column chromatography gave 248 mg of the title compound which was used in the next step without further characterization.

Step 2. 7-Bromo-3-(pyrrolidin-3-yl)isoquinolin-1(2*H*)-one (**21**): To a solution of *tert*-butyl 3-(7-bromo-1-oxo-1,2-dihydroisoquinolin-3-yl)pyrrolidine-1-carboxylate (**20**; 248 mg) in DCM (12 mL) was added TFA (0.97 mL, 12.6 mmol) at room temperature. After 2 h, the solvent was removed, and the crude product (360 mg) was used in the next step without prior purification.

Step 3. 7-Bromo-3-[1-(prop-2-enoyl)pyrrolidin-3-yl]isoquinolin-1(2*H*)-one (**22**): To a solution of 7-bromo-3-(pyrrolidin-3-yl)isoquinolin-1(2*H*)-one (**21**; 360 mg) in DCM (40 mL) were added prop-2-enoyl chloride (133 mg, 1.47 mmol) and *N*,*N*-diisopropylethylamine (2.2 mL, 12.3 mmol). The reaction mixture was allowed to stir at room temperature overnight. Then, the mixture was diluted with saturated aqueous $NaHCO_3$ solution and extracted with DCM. The combined organic phases were washed with saturated aqueous NaCl solution and dried over Na_2SO_4. Filtration, removal of the solvent, and column chromatography gave 222 mg of the title compound which was used without further characterization.

Step 4. 7-(2-Fluoro-6-hydroxyphenyl)-3-[1-(prop-2-enoyl)pyrrolidin-3-yl]isoquinolin-1(2*H*)-one (**2**): To a solution of 7-bromo-3-[1-(prop-2-enoyl)pyrrolidin-3-yl]isoquinolin-1(2*H*)-one (**22**; 85 mg, 0.245 mmol) in 1,4-dioxane/water (7.5 mL/2.2 mL) in a sealed tube were added (2-fluoro-6-hydroxyphenyl)boronic acid (42 mg, 0.27 mmol), dicyclohexyl(2',6'-diisopropoxy-1,1'-biphenyl-2-yl)phosphine (RuPhos; 11 mg, 0.024 mmol), (2-dicyclohexylphosphino-2′,6′-diisopropoxy-1,1′-biphenyl)[2-(2′-amino-1,1′-biphenyl)]palladium(II) methanesulfonate (RuPhos Pd G3; 10 mg, 0.012 mmol), and K_2CO_3 (85 mg, 0.612 mmol). The reaction mixture was stirred at 65 °C for 18 h. After cooling to room temperature, the reaction mixture was diluted with saturated aqueous NH_4Cl solution, then extracted with EtOAc. The combined organic phases were dried over Na_2SO_4. Filtration, removal of the solvent, and purification (HPLC Method 1) of the crude gave 14 mg (15% yield) of the title compound as a mixture of rotamers. ^{1}H NMR (400 MHz, [D$_6$]DMSO): δ [ppm] = 2.02–2.40 (m, 4H), 3.37–3.48 (m, 4H), 3.57–3.69 (m, 3H), 3.73–3.82 (m, 1H), 3.87–3.95 (m, 1H), 4.03–4.10 (m, 1H), 5.67–5.73 (m, 2H), 6.13–6.21 (m, 2H), 6.50 (d, *J*=10.7 Hz, 2H), 6.58–6.66 (m, 2H), 6.71–6.77 (m, 2H), 6.82 (d, *J*=9.3 Hz, 2H), 7.17–7.26 (m, 2H), 7.54–7.75 (m, 4H), 8.14 (s, 2H), 9.94–10.08 (br, 2H), 11.30–11.43 (m, 2H); MS (ESI+): *m*/*z* = 379 $[M+H]^+$; LC-MS (Method 1): t_R = 0.82, 0.85 min.

2.3.3 7-(2,4-Difluorophenyl)-3-[1-(prop-2-enoyl)pyrrolidin-3-yl]isoquinolin-1(2*H*)-one (**3**)

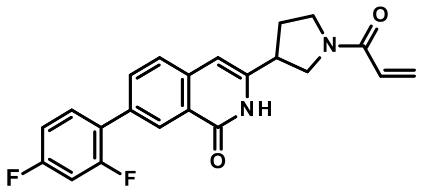

Starting from 7-bromo-3-[1-(prop-2-enoyl)pyrrolidin-3-yl]isoquinolin-1(2*H*)-one (**22**; 97 mg, 0.279 mmol), the title compound was prepared in an analogous fashion as described for compound **2** and was obtained as a mixture of rotamers (14 mg, 13% yield) after purification (HPLC Method 2). ^{1}H NMR (400 MHz, [D$_6$]DMSO): δ [ppm] = 2.03–2.40 (m, 4H), 3.36–3.49 (m, 4H), 3.59–3.68 (m, 3H), 3.73–3.81 (m, 1H), 3.87–3.95 (m, 1H), 4.02–4.09 (m, 1H), 5.65–5.73 (m, 2H), 6.11–6.21 (m, 2H), 6.53 (d, *J*=9.4 Hz, 2H), 6.56–6.68 (m, 2H), 7.19–7.27 (m, 2H), 7.37–7.47 (m, 2H), 7.65–7.74 (m, 4H), 7.79–7.87 (m, 2H), 8.27 (s, 2H), 11.37–11.51 (br, 2H); MS (ESI+): *m*/*z* = 381 [*M*+H]$^+$; LC-MS (Method 1): t_R = 1.03 min.

2.3.4 7-(3,5-Difluorophenyl)-3-[1-(prop-2-enoyl)pyrrolidin-3-yl]isoquinolin-1(2*H*)-one (**4**)

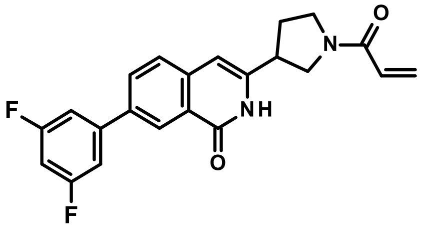

Starting from 7-bromo-3-[1-(prop-2-enoyl)pyrrolidin-3-yl]isoquinolin-1(2*H*)-one (**22**; 97 mg, 0.279 mmol), the title compound was prepared in an analogous fashion as described for compound **2** and was obtained as a mixture of rotamers (17 mg, 16% yield) after purification (HPLC Method 2). ^{1}H NMR (400 MHz, [D$_6$]DMSO): δ [ppm] = 1.99–2.40 (m, 4H), 3.36–3.48 (m, 4H), 3.58–3.68 (m, 3H), 3.75–3.82 (m, 1H), 3.88–3.95 (m, 1H), 4.03–4.09 (m, 1H), 5.59–5.75 (m, 2H), 6.10–6.24 (m, 2H), 6.53 (d, *J*=8.11 Hz, 2H), 6.57–6.66 (m, 2H), 7.19–7.34 (m, 2H), 7.43–7.61 (m, 4H), 7.72 (dd, *J*=8.36, 3.80 Hz, 2H), 8.04–8.08 (m, 2H), 8.42 (s, 2H), 11.26–11.63 (br, 2H); MS (ESI+): *m*/*z* = 381 [*M*+H]$^+$; LC-MS (Method 1): t_R = 1.05 min.

2.3.5 7-(2-Ethylphenyl)-3-[1-(prop-2-enoyl)pyrrolidin-3-yl]isoquinolin-1(2*H*)-one (**5**)

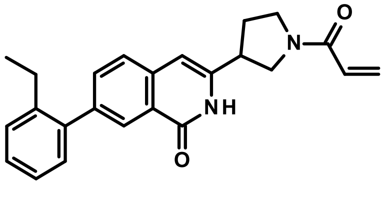

Starting from 7-bromo-3-[1-(prop-2-enoyl)pyrrolidin-3-yl]isoquinolin-1(2*H*)-one (**22**; 97 mg, 0.279 mmol), the title compound was prepared in an analogous fashion as described for compound **2** and was obtained as a mixture of rotamers (21 mg, 20% yield) after purification (HPLC Method 2). ^{1}H NMR (400 MHz, [D$_6$]DMSO): δ [ppm] = 1.02 (t, *J*=7.60 Hz, 6H), 2.03–2.41 (m, 4H), 2.56 (q, *J*=7.44 Hz, 4H), 3.36–3.51 (m, 4H), 3.57–3.69 (m, 3H), 3.74–3.84 (m, 1H), 3.88–3.96 (m, 1H), 4.01–4.11 (m, 1H), 5.64–5.79 (m, 2H), 6.10–6.24 (m, 2H), 6.53 (d, *J*=10.14 Hz, 2H), 6.57–6.68 (m, 2H), 7.14–7.23 (m, 2H), 7.24–7.31 (m, 2H), 7.33–7.40 (m, 4H), 7.59–7.73 (m, 4H), 8.02 (s, 2H), 11.30–11.49 (br, 2H).

2.3.6 3-[1-(Prop-2-enoyl)pyrrolidin-3-yl]-7-(quinolin-5-yl)isoquinolin-1(2*H*)-one (**6**)

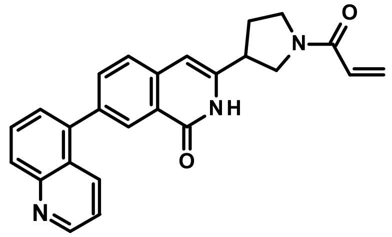

Starting from 7-bromo-3-[1-(prop-2-enoyl)pyrrolidin-3-yl]isoquinolin-1(2*H*)-one (**22**; 97 mg, 0.279 mmol), the title compound was prepared in an analogous fashion as described for compound **2** and was obtained as a mixture of rotamers (21 mg, 20% yield) after purification (HPLC Method 2). ^{1}H NMR (400 MHz, [D$_6$]DMSO): δ [ppm] = 2.04–2.43 (m, 4H), 3.38–3.53 (m, 4H), 3.58–3.72 (m, 3H), 3.75–3.86 (m, 1H), 3.89–3.96 (m, 1H), 4.03–4.13 (m, 1H), 5.63–5.76 (m, 2H), 6.11–6.24 (m, 2H), 6.55–6.69 (m, 4H), 7.49–7.58 (m, 2H), 7.61–7.66 (m, 2H), 7.74–7.91 (m, 6H), 8.05–8.15 (m, 2H), 8.16–8.28 (m, 4H), 8.86–9.01 (m, 2H), 11.20–11.60 (br, 2H); MS (ESI+): *m*/*z* = 396 [*M*+H]$^+$; LC-MS (Method 1): t_R = 0.69 min.

2.3.7 6-Chloro-7-(2-fluoro-6-hydroxyphenyl)-3-[1-(prop-2-enoyl)pyrrolidin-3-yl]isoquinolin-1(2*H*)-one (**7**)

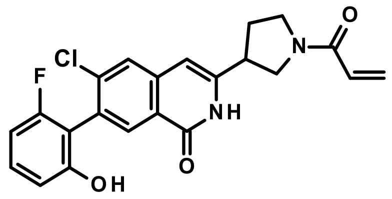

Step 1. 2,5-Dibromo-4-chlorobenzoic acid: To a solution of 2-bromo-4-chlorobenzoic acid (500 mg, 2.12 mmol) in chlorosulfonic acid (1.41 mL, 21.24 mmol) were added, at room temperature, sulfur (5.2 mg, 0.16 mmol) and bromine (109 µL, 2.12 mmol). The reaction mixture was stirred overnight at 70 °C, then cooled to room temperature. Water was cautiously added to the mixture. After stirring for 30 min, the precipitates were collected by filtration and purified by column chromatography to give 512 mg of the title compound which was used in the next step without prior characterization.

Step 2. 5-Bromo-4-chloro-2-(2-ethoxy-2-oxoethyl)benzoic acid: To a solution of 2,5-dibromo-4-chlorobenzoic acid (512 mg, 1.63 mmol) and ethyl 3-oxobutanoate (424 mg, 3.26 mmol) in EtOH (10 mL) were added, at room temperature, CuBr (234 mg, 1.63 mmol) and sodium ethoxide (1.82 mL, 4.89 mmol). The mixture was refluxed for 2 h. After cooling to room temperature, the mixture was acidified with 2 N HCl and the solvent was removed under reduced pressure. Water was added, followed by extraction with DCM. The combined organic phases were dried over Na_2SO_4. Filtration, removal of the solvent, and subsequent purification by column chromatography gave 452 mg (66% yield over 2 steps) of the title compound. ^{1}H NMR (500 MHz, [D$_6$]DMSO): δ [ppm] = 1.16 (t, *J*=7.15 Hz, 3H), 3.97 (s, 2H), 4.06 (q, *J*=6.99 Hz, 2H), 7.69 (s, 1H), 8.17 (s, 1H).

Step 3. 5-Bromo-2-(carboxymethyl)-4-chlorobenzoic acid: To a solution of 5-bromo-4-chloro-2-(2-ethoxy-2-oxoethyl)benzoic acid (359 mg, 1.17 mmol) in a MeOH/THF mixture (4 mL/11 mL) was added 2 N NaOH solution (5.0 mL, 10.0 mmol). After 2 h at room temperature, the solvent was removed. The crude was taken up in water and acidified with concd HCl at 0 °C. After a further 30

min, the precipitates were collected by filtration, dried overnight, and used in the next step without prior purification or characterization (240 mg).

Step 4. 7-Bromo-6-chloroisochromane-1,3-dione: To a solution of 5-bromo-2-(carboxymethyl)-4-chlorobenzoic acid (210 mg) in acetonitrile (4.0 mL) was added acetyl chloride (3.8 mL, 4.29 mmol). The reaction mixture was stirred for 3 h at 50 °C, then cooled to room temperature. The solvent was removed under reduced pressure and the crude (220 mg) was used in the next step without prior purification or characterization.

Step 5. *tert*-Butyl 3-(7-bromo-6-chloro-1-oxo-1,2-dihydroisoquinolin-3-yl)pyrrolidine-1-carboxylate: Prepared from 7-bromo-6-chloroisochromane-1,3-dione in an analogous fashion as described for compound **20** to give 200 mg of the title compound which was used without prior characterization.

Step 6. 7-Bromo-6-chloro-3-(pyrrolidin-3-yl)isoquinolin-1(2*H*)-one: Prepared from *tert*-butyl 3-(7-bromo-6-chloro-1-oxo-1,2-dihydroisoquinolin-3-yl)pyrrolidine-1-carboxylate in an analogous fashion as described for compound **21** to give 200 mg of the title compound which was used without prior characterization.

Step 7. 7-Bromo-6-chloro-3-[1-(prop-2-enoyl)pyrrolidin-3-yl]isoquinolin-1(2*H*)-one: Prepared from 7-bromo-6-chloro-3-(pyrrolidin-3-yl)isoquinolin-1(2*H*)-one in an analogous fashion as described for compound **22** to give 60 mg of the title compound which was used without prior characterization.

Step 8. 6-Chloro-7-(2-fluoro-6-hydroxyphenyl)-3-[1-(prop-2-enoyl)pyrrolidin-3-yl]isoquinolin-1(2*H*)-one (**7**): Prepared from 7-bromo-6-chloro-3-[1-(prop-2-enoyl)pyrrolidin-3-yl]isoquinolin-1(2*H*)-one in an analogous fashion as described for compound **2** to give 6 mg (8% yield, for the final step) of the title compound. MS (ESI+): m/z = 413 $[M+H]^+$; LC-MS (Method 1): t_R = 0.92 min.

2.3.8 6-(2-Fluoro-6-hydroxyphenyl)-2-[1-(prop-2-enoyl)pyrrolidin-3-yl]quinazolin-4(3*H*)-one (**11**)

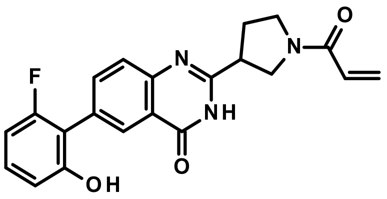

Step 1. *tert*-Butyl 3-[(4-bromo-2-carbamoylphenyl)carbamoyl]pyrrolidine-1-carboxylate: To a solution of 2-amino-5-bromobenzamide (2.0 g, 9.3 mmol) and 1-(*tert*-butoxycarbonyl)pyrrolidine-3-carboxylic acid (2.0 g, 9.3 mmol) in *N*,*N*-dimethylformamide (20 mL) were added HATU (3.89 g, 10.23 mmol) and *N*,*N*-diisopropylethylamine (4.86 mL, 28 mmol). The reaction mixture was stirred at 80 °C overnight. Addition of water (200 µL) and removal of solvent, followed by column chromatography, gave 2.99 g of the title compound which was used in the next step without prior characterization.

Step 2. *tert*-Butyl 3-(6-bromo-4-oxo-3,4-dihydroquinazolin-2-yl)pyrrolidine-1-carboxylate: To a solution of *tert*-butyl 3-[(4-bromo-2-carbamoylphenyl)carbamoyl]pyrrolidine-1-carboxylate (2.99 g, 7.25 mmol) in MeOH (150 mL) was added 30% methanolic sodium methoxide (2.7 mL, 15 mmol). The reaction mixture was stirred at 50 °C for 2 h, then cooled to room temperature. Removal of the solvent gave the crude product which was dissolved in EtOAc and washed with water. The organic phase was dried over Na_2SO_4. Filtration, removal of the solvent, and chromatography gave 1.15 g of the title compound which was used in the next step without prior characterization.

Step 3. 6-Bromo-2-(pyrrolidin-3-yl)quinazolin-4(3*H*)-one: To a solution of *tert*-butyl 3-(6-bromo-4-oxo-3,4-dihydroquinazolin-2-yl)pyrrolidine-1-carboxylate (1.15 g, 2.92 mmol) in THF (12 mL) was added 4 M HCl in dioxane (45 mL). The reaction mixture was stirred for 3 h at room temperature, followed by 1 h at 60 °C. After completion of the reaction, the solvent was removed and the crude product (858 mg) was used in the next step without prior purification.

Step 4. 6-Bromo-2-[(1-(prop-2-enoyl)pyrrolidin-3-yl]quinazolin-4(3*H*)-one: Starting from 6-bromo-2-(pyrrolidin-3-yl)quinazolin-4(3*H*)-one, the title compound was prepared in an analogous fashion as described for compound **22** to give 524 mg (15% overall yield over 4 steps). ^{1}H NMR (400 MHz, [D$_6$]DMSO): δ [ppm] = 2.09–2.40 (m, 2H), 3.35–3.85 (m, 4H), 3.88–3.99 (m, 1H), 5.68 (dd, *J*=10.27, 2.41 Hz, 1H), 6.15 (dd, *J*=16.86, 2.41 Hz, 1H), 6.55–6.66 (m, 1H), 7.56 (dd, *J*=8.74, 1.65 Hz, 1H), 7.93 (ddd, *J*=8.74, 2.41, 1.01 Hz, 1H), 8.17 (dd, *J*=2.28, 1.27 Hz, 1H), 12.51 (br, 1H); MS (ESI+): *m*/*z* = 348 [*M*+H]$^+$; LC-MS (Method 1): t_R = 0.83 min.

Step 5. 6-(2-Fluoro-6-hydroxyphenyl)-2-[1-(prop-2-enoyl)pyrrolidin-3-yl]quinazolin-4(3*H*)-one (**11**): Prepared from 6-bromo-2-[(1-(prop-2-enoyl)pyrrolidin-3-yl]quinazolin-4(3*H*)-one (105 mg, 0.302 mmol) in an analogous fashion as described for compound **2** to give 22 mg (19% yield) of the title compound as a mixture of atropisomers after purification (HPLC Method 1). ^{1}H NMR (500 MHz, [D$_6$]DMSO, 80 °C): δ [ppm] = 2.17–2.44 (m, 4H), 3.38–4.08 (m, 10H), 5.58–5.71 (m, 2H), 6.06–6.20 (m, 2H), 6.47–6.67 (m, 2H), 6.68–6.78 (m, 2H), 6.84 (d, *J*=8.27 Hz, 2H), 7.11–7.28 (m, 2H), 7.55 (d, *J*=8.58 Hz, 1H), 7.63 (d, *J*=8.58 Hz, 1H), 7.76–7.79 (m, 1H), 7.90 (dd, *J*=8.58, 2.54 Hz, 1H), 8.11 (s, 1H), 8.18 (m, 1H), 9.73 (br, 2H), 12.10 (br, 2H).

2.3.9 7-(2-Fluoro-6-hydroxyphenyl)-3-[1-(prop-2-enoyl)pyrrolidin-3-yl]-2,6-naphthyridin-1(2*H*)-one (**13**)

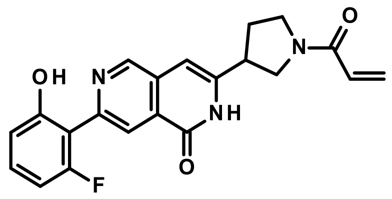

Step 1. Ethyl 5-bromo-2-chloropyridine-4-carboxylate: 5-Bromo-2-chloropyridine-4-carboxylic acid (2.00 g, 8.46 mmol) was dissolved in EtOH (40 mL), and sulfuryl chloride (690 µL, 8.5 mmol) was added. The obtained mixture was stirred at reflux for 17 h. Then, the mixture was concentrated under reduced pressure and poured into ice-cold water. The cold solution was extracted with EtOAc. The combined organic layers were washed with water and saturated aqueous Na_2CO_3 solution, then dried over Na_2SO_4 and concentrated to give 2.18 g (97% yield) of the title compound. ^{1}H NMR (400 MHz, [D$_6$]DMSO): δ [ppm] = 1.33 (t, *J*=7.10 Hz, 3H), 4.37 (q, *J*=7.10 Hz, 2H), 7.89 (s, 1H), 8.77 (s, 1H); MS (ESI+): *m*/*z* = 265 [*M*+H]$^+$; LC-MS (Method 2): t_R = 1.20 min.

Step 2. Ethyl 5-{[1-(*tert*-butoxycarbonyl)pyrrolidin-3-yl]ethynyl}-2-chloropyridine-4-carboxylate: Ethyl 5-bromo-2-chloropyridine-4-carboxylate (542 mg, 2.05 mmol) was suspended in THF (11 mL). CuI (19.5 mg, 102 µmol) and bis(triphenylphosphine)palladium(II) dichloride (71.9 mg, 102 µmol) were added, and the reaction vessel was flushed with nitrogen. Triethylamine (621 mg, 6.15 mmol) and *tert*-butyl 3-ethynylpyrrolidine-1-carboxylate (480 mg, 2.46 mmol) were added. The vessel was flushed again with nitrogen and the mixture was stirred at reflux for 15 h. The reaction mixture was cooled to room temperature and poured into saturated aqueous $NaHCO_3$ solution. The aqueous layer was extracted with EtOAc, and the combined extracts were washed with brine and dried over Na_2SO_4. The organic layer was concentrated and the obtained crude product was purified by flash chromatography to give 563 mg (73% yield) of the title compound. ^{1}H NMR (400 MHz, [D$_6$]DMSO): δ [ppm] = 1.33 (t, *J*=7.10 Hz, 3H), 1.46 (s, 9H), 1.85–1.96 (m,

1H), 2.14–2.24 (m, 1H), 3.19–3.31 (m, 2H), 3.36–3.45 (m, 2H), 3.54–3.65 (m, 1H), 4.35 (q, *J*=7.10 Hz, 2H), 7.84 (s, 1H), 8.62 (s, 1H); MS (ESI+): *m*/*z* = 379 $[M+H]^+$; LC-MS (Method 2): t_R = 1.45 min.

Step 3. 5-{[1-(*tert*-Butoxycarbonyl)pyrrolidin-3-yl]ethynyl}-2-chloropyridine-4-carboxylic acid: Ethyl 5-{[1-(*tert*-butoxycarbonyl)pyrrolidin-3-yl]ethynyl}-2-chloropyridine-4-carboxylate (516 mg, 1.36 mmol) was dissolved in MeOH (3.0 mL) and 1.0 M aqueous NaOH (2.0 mL, 2.0 mmol) was added. The obtained mixture was stirred at room temperature for 1 h. The reaction mixture was diluted with water and the pH adjusted to 4.0 using 1 M HCl solution. The aqueous layer was extracted with EtOAc. The combined organic layers were dried over Na_2SO_4 and concentrated. The crude product was purified by flash chromatography to give 571 mg (83% purity, quantitative yield) of the title compound which was used without further purification. MS (ESI–): *m*/*z* = 349 $[M–H]^+$; LC-MS (Method 2): t_R = 1.15 min.

Step 4. 5-{[1-(*tert*-Butoxycarbonyl)pyrrolidin-3-yl]ethynyl}-2-(2-fluoro-6-methoxyphenyl)pyridine-4-carboxylic acid: 5-{[1-(*tert*-Butoxycarbonyl)pyrrolidin-3-yl]ethynyl}-2-chloropyridine-4-carboxylic acid (440 mg, 1.04 mmol) was suspended in 1,4-dioxane (44 mL). (2-Fluoro-6-methoxyphenyl)boronic acid (320 mg, 1.88 mmol), $Pd(PPh_3)_4$ (72.5 mg, 62.7 µmol), and 2.0 M aqueous Na_2CO_3 solution (1.9 mL, 3.8 mmol) were added. The mixture was stirred at 100 °C for 24 h, then cooled to room temperature. Saturated aqueous NH_4Cl solution was added, and the obtained mixture was extracted with EtOAc. The combined organic layers were dried over Na_2SO_4 and concentrated. The crude product was purified by flash chromatography to give 81 mg (17% yield) of the title compound. ^1H NMR (400 MHz, $[D_6]$DMSO): δ [ppm] = 1.42 (s, 9H), 1.98–2.28 (m, 2H), 3.35–3.70 (m, 5H), 3.75 (s, 3H), 6.83 (s, 1H), 6.89–6.98 (m, 1H), 7.03 (d, *J*=8.36 Hz, 1H), 7.43–7.53 (m, 1H), 7.94 (s, 1H), 9.07 (s, 1H); MS (ESI+): *m*/*z* = 441 $[M+H]^+$; LC-MS (Method 2): t_R = 1.31 min.

Step 5. 7-(2-Fluoro-6-methoxyphenyl)-3-(pyrrolidin-3-yl)-1*H*-pyrano[4,3-*c*]pyridin-1-one: 5-{[1-(*tert*-Butoxycarbonyl)pyrrolidin-3-yl]ethynyl}-2-(2-fluoro-6-methoxyphenyl)pyridine-4-carboxylic acid (60.0 mg, 136 µmol) was dissolved in DCM (3.0 mL) and THF (3.0 mL). Trifluoromethane-sulfonic acid (18 µL, 200 µmol) was slowly added at 0 °C. The suspension was stirred at room temperature for 17 h. DCM was added, and the mixture was concentrated under reduced pressure to give 46.3 mg (quantitative yield) of a residue which was used directly in the next step without further purification.

Step 6. 7-(2-Fluoro-6-methoxyphenyl)-3-(pyrrolidin-3-yl)-2,6-naphthyridin-1(2*H*)-one: 7-(2-Fluoro-6-methoxyphenyl)-3-(pyrrolidin-3-yl)-1*H*-pyrano[4,3-*c*]pyridin-1-one (46.3 mg, 136 µmol) was dissolved in 7.0 M methanolic ammonia (3.0 mL, 21 mmol). The suspension was stirred at 69 °C for 4 d. The mixture was concentrated under reduced pressure to give 98.0 mg of a residue which was used directly in the next step without further purification. MS (ESI+): *m*/*z* = 340 $[M+H]^+$; LC-MS (Method 2): t_R = 0.63 min.

Step 7. 7-(2-Fluoro-6-methoxyphenyl)-3-[1-(prop-2-enoyl)pyrrolidin-3-yl]-2,6-naphthyridin-1(2*H*)-one: 7-(2-Fluoro-6-methoxyphenyl)-3-(pyrrolidin-3-yl)-2,6-naphthyridin-1(2*H*)-one (46.2 mg, 136 µmol) was suspended in DCM (4.0 mL) and triethylamine (95 µL, 680 µmol) was added. prop-2-enoyl chloride (13 µL, 160 µmol) in DCM (100 µL) was added. The mixture was stirred at room temperature for 1 h. Saturated aqueous Na_2CO_3 solution was added at 0 °C, and the obtained mixture was extracted with EtOAc. The combined organic layers were dried over Na_2SO_4 and concentrated. The crude product was purified by chromatography to give 35.5 mg (95% purity, 63% yield) of the title compound as a mixture of rotamers. MS (ESI–): *m*/*z* = 392 $[M–H]^–$; LC-MS (Method 2): t_R = 0.84 min.

Step 8. 7-(2-Fluoro-6-hydroxyphenyl)-3-[1-(prop-2-enoyl)pyrrolidin-3-yl]-2,6-naphthyridin-1(2*H*)-one (**13**): 7-(2-Fluoro-6-methoxyphenyl)-3-[1-(prop-2-enoyl)pyrrolidin-3-yl]-2,6-naphthyridin-1(2*H*)-one (32.2 mg, 81.8 µmol) was dissolved in DCM (4.8 mL). At 0 °C, 1.0 M boron tribromide

in DCM (820 µL, 820 µmol) was slowly added. The reaction mixture was stirred at room temperature for 17 h. Saturated aqueous Na_2CO_3 solution was added at 0 °C, and the obtained mixture was extracted with EtOAc. The combined organic layers were dried over Na_2SO_4 and concentrated. The crude product was purified by flash chromatography to give 24.0 mg (94% purity, 73% yield) of the title compound. ^{1}H NMR (500 MHz, [D$_6$]DMSO): δ [ppm] = 2.03–2.42 (m, 2H), 3.40–3.53 (m, 2H), 3.61–3.70 (m, 2H), 3.80–4.12 (m, 1H), 5.65–5.75 (m, 1H), 6.10–6.23 (m, 1H), 6.65–6.71 (m, 2H), 6.75–6.86 (m, 2H), 7.25–7.33 (m, 1H), 8.43 (s, 1H), 9.12 (s, 1H), 11.85 (br d, *J*=12.40 Hz, 1H), 12.58 (d, *J*=5.40 Hz, 1H); MS (ESI–): *m*/*z* = 378 [*M*–H]$^-$; LC-MS (Method 2): t_R = 0.98 min.

3. Protein production, crystallization, and mass spectrometry

3.1 Protein preparation

The recombinant proteins KRAS P01116-2 and SOS1 Q07889 were produced as described in a protocol published recently.[19] The proteins KRAS^{WT} (aa 1–169, wild type), SOS1^{CAT} (aa 564–1049, wild type), and KRAS^{G12C} (aa 1–169, G12C) were produced for the biochemical assay. KRAS^{G12C_SB2} (aa 1–169; G12C, C51S, C80L, C118S) was used for crystallization with covalently bound compounds, and its protein production was performed as described previously for KRAS^{G12C_SB}.[19]

Co-crystallization of KRAS with covalent ligands was conducted by incubating 50 µM tag-free recombinant KRAS^{G12C_SB2} with 200 µM of covalent ligand in 50 mM Tris, pH 8, 50 mM NaCl (1% v/v DMSO final concentration) for 60 min at 37 °C. Free ligand was removed by using a desalting column (HiPrep 26/10) on an ÄKTA pure system (GE Life Sciences) and a running buffer of 20 mM HEPES, pH 7.5, 150 mM NaCl, 1 mM $MgCl_2$. The protein–ligand complex was concentrated to >12 mg/mL using a centrifugal concentrator (Amicon, 10 kDa cutoff). The protein concentration was determined by absorbance at 280 nm using a NanoDrop spectrophotometer (ThermoFisher Scientific). The percentage of modified KRAS was determined by LC-MS as described in Section 3.4 and was, in most cases, >90%. The freshly prepared protein–ligand complex was used for crystallization, without freezing.

3.2 Crystallography

3.2.1 Compound **3**

Covalently modified protein (>12 mg/mL) crystallized in 100 mM MES, pH 6.9, and 34% PEG 4000. Protein (100 nL) was mixed with reservoir solution (100 nL) and incubated at 20 °C in sitting drop crystallization plates (Intelli-Plate 96, Art Robbins Instruments). 20% Glycerol was used for cryo-protection of crystals prior to freezing in liquid nitrogen. The crystal structure has been deposited at the RCSB PDB (PDB accession code 6TAM). In comparison to the crystal structure with ARS-1620 (PDB entry 5V9U),[10] only some differences are detected in Switch-II and the position of the subsequent following Helix-2. These differences are likely due to the ligand bound in the induced allosteric pocket underneath.

3.2.2 Compound **13**

Covalently modified protein (18.8 mg/mL) crystallized in 100 mM MES, pH 6.3, and 31% PEG 4000. Protein (100 nL) was mixed with reservoir solution (100 nL) and incubated at 20 °C in sitting drop crystallization plates (Intelli-Plate 96, Art Robbins Instruments). The crystal was frozen without any cryo buffer. The crystal structure has been deposited at the RCSB PDB (PDB accession code 6TAN).

3.2.3 Data collection and refinement

Diffraction data were collected using synchrotron radiation at BESSY II/BL 14.1 (Helmholtz-Zentrum Berlin, Germany). The data were processed in XDS/XDSAPP,[20] phased by molecular replacement in Phaser,[21] and further refined in REFMAC5[21b, 22] and Coot.[23] Compound parameterization was performed in PRODRG [24] and further modified to allow a covalent bond to Cys12 with JLigand,[25] as implemented in Coot.[23] Data quality and refinement statistics are summarized in Table S1. The compound position and conformation were completely defined by electron density. Switch-II exhibits additional conformations in the crystal, leading to high B-factors for these residues (Ala59–Ser65).

Table S1. Diffraction data quality and refinement statistics.

	KRAS^{G12C_SB2} + Compound 3	KRAS^{G12C_SB2} + Compound 13
**PDB Accession Code**	6TAM	6TAN
**Data Collection**		
Beamline[1]	BESSY II, BL14.1	BESSY II, BL14.1
Wavelength [Å]	0.979500	0.918409
Space group (No.)	$P2_12_12_1$ (19)	$P2_12_12_1$ (19)
Unit cell, *a*, *b*, *c* [Å]	41.1, 58.5, 66.1	41.3, 58.8, 65.6
Resolution limit [Å][2]	50.0–1.64 (1.74–1.64)	43.8–1.16 (1.16–1.23)
No. of reflections[2]	256609 (36223)	400635 (59295)
No. of unique reflections[2]	20063 (3072)	55595 (8759)
Multiplicity[2]	12.8 (11.8)	7.2 (6.8)
Completeness [%][2]	99.2 (95.3)	99.1 (98.1)
$I/\sigma(I)$[2]	20.1 (2.17)	13.7 (1.2)
R_{meas} [%][2]	7.1 (99.1)	6.5 (153.1)
CC(1/2) [%][2]	99.9 (81.8)	99.9 (52.2)
B (Wilson) [Å^2]	34.2	19.0
Mosaicity [deg]	0.183	0.106
**Refinement**		
Resolution limit [Å][2]	43.83–1.64 (1.682–1.640)	43.77–1.16 (1.189–1.159)
Completeness [%][2]	99.23 (89.59)	99.07 (97.40)
No. of reflections[2]	19061 (1247)	53494
R_{work}/R_{free} [%][2]	17.42/20.05 (29.0/26.9)	13.93/18.34 (31.3/31.9)
Mean *B* value [Å^2]	27.4	20.8
rmsd bond length [Å]	0.007	0.011
rmsd bond angles [deg]	1.48	1.71

[1] Synchrotron: BESSY II, Helmholtz-Zentrum Berlin, Germany.
[2] Values in brackets refer to the highest resolution shell.

3.3 Biochemical activity assays

Measurement of (i) KRAS^{G12C} activation by SOS1^{CAT} and (ii) wild-type KRAS activation by SOS1^{CAT} was performed following the previously described protocol,[19] with a pre-incubation time of the test compound with KRAS proteins extended to 180 min before addition of SOS1.

This assay quantifies SOS1^{CAT} mediated loading of KRAS^{G12C}–GDP with a fluorescent GTP analogue. Detection of successful loading was achieved by measuring resonance energy transfer from anti-GST-terbium (FRET donor) bound to GST-KRAS^{G12C} to the loaded fluorescent GTP analogue (FRET acceptor). The fluorescent GTP analogue EDA–GTP–DY-647P1 [2'/3'-O-(2-aminoethyl-carbamoyl)guanosine-5'-triphosphate labelled with DY-647P1 (Dyomics GmbH, Germany)] was synthesized by Jena Bioscience (Germany) and supplied as a 1 mM aqueous solution. A KRAS^{G12C} working solution was prepared in assay buffer [10 mM HEPES pH 7.4 (AppliChem), 150 mM NaCl (Sigma), 5 mM MgCl2 (Sigma), 1 mM DTT (Thermo Fisher), 0.05% BSA Fraction V pH 7.0 (ICN Biomedicals), 0.0025% (v/v) Igepal (Sigma)] containing 100 nM GST-KRAS^{G12C} and 2 nM anti-GST-terbium (Cisbio, France). A SOS1^{CAT} working solution was prepared in an assay buffer containing 20 nM SOS1^{CAT} and 200 nM EDA–GTP–DY-647P1. An inhibitor control solution was prepared in an assay buffer containing 200 nM EDA–GTP–DY-647P1 without

SOS1^{CAT}. All steps of the assay were performed at 20 °C. A volume of 2.5 µL of the KRAS^{G12C} working solution was added to all wells of the test plate using a Multidrop dispenser (Thermo LabSystems). After 180 min, 2.5 µL of the SOS1^{CAT} working solution was added to all wells, except for the inhibitor control solution wells. After 30 min incubation, HTRF was measured.

In the KRAS^{WT} activation by SOS1cat assay, human SOS1^{CAT} mediated loading of GST-KRAS^{WT}–GDP was quantified with a fluorescent GTP analogue. The assay was performed as described above for the KRAS^{G12C} activation. GST-KRAS^{G12C} was replaced by GST-KRAS^{WT}, used at a final concentration of 50 nM.

3.4 Covalent binding assay by mass spectrometry

The percentage of covalent adduct formation at KRAS^{G12C} was determined by intact mass determination. To this end, 25 µM recombinant KRAS mutant (G12C, C51S, C80L, C118S) was incubated with 25 µM compound (1% v/v DMSO final concentration) in 50 mM Tris, pH 8.0, 50 mM NaCl at room temperature for 2 h. Reaction mixture (20 µL) was acidified by addition of 4% v/v TFA (4 µL). LC-MS analysis was performed using a Waters SYNAPT G2-S quadrupole time-of-flight mass spectrometer connected to a Waters nanoACQUITY UPLC system. Samples were loaded onto a 2.1 × 5 mm MassPrep C4 guard column (Waters) and desalted with a short gradient (3 min) of increasing concentration of acetonitrile at a flow rate of 100 µL/min. Spectra were analyzed by using MassLynx v4.1 software and deconvoluted with the MaxEnt1 algorithm. Percent conversion was determined by the ratio of signal intensities of KRAS^{WT} and KRAS^{G12C} + inhibitor.

3.5 Cellular assay for RAS activation

Using the G-LISA Kit (Cytoskeleton BK131, RAS Activation Assay), 50000 cells are seeded in a 96 well plate for 72 h at 37 °C (10% FBS, DMEM/Ham's F-12, 2 mM L-Glutamine). Cells are treated with varying concentrations of test compounds for 24 hours, and then treated with the lysis buffer. All next steps are performed according to the supplier's manual, at 0 °C (https://www.cytoskeleton.com/bk131). Finally, the content of active RAS is measured by detecting the absorbance at 490 nm. The value of untreated cells is set as 100%, whereas the value of the blank is set as 0%. The resulting percentage reflects the level of inhibition of the active RAS formation compared to the control. All compounds reported in this manuscript showed no activity in this assay, except for compound **13** (IC_{50} = 22 µM).

3.6 Determination of k_{inact} and K_I

The percentage of covalent adduct formation at KRAS^{G12C} was determined for compounds **1**, **2** and **13** by intact mass determination. To this end, 25 µM of the same recombinant KRAS mutant as for the covalent binding assay (G12C, C51S, C80L, C118S) were incubated with 25 µM of compound (1% v/v final DMSO concentration) in 50 mM Tris, pH8, 50 mM NaCl at room temperature for 2 h. The reaction was acidified by adding 4 µL of 4% v/v TFA to 20 µL reaction volume.

Liquid chromatography-mass spectrometry (LC-MS) analysis was performed using a Waters SYNAPT G2-S quadrupole time-of-flight mass spectrometer connected to a Waters nanoAcquity UPLC system. Samples were loaded on a 2.1 x 5 mm mass prep C4 guard column (Waters) and desalted with a short gradient (3 min) of increasing concentrations of acetonitrile at a flow rate of 100 µL/min. Spectra were analyzed by using MassLynx v4.1 software and deconvoluted with the MaxEnt1 algorithm. Percent of conversion was determined by the ratio of signal intensities of apo-KRAS and KRAS + inhibitor.

The k_{inact} / K_I ratio was determined by measuring the total occupancy (% conversion) over time at different inhibitor concentrations. In detail, 10 µM KRAS^{G12C} were incubated in the same reaction buffer as above with 10 µM, 25 µM, 50 µM or 100 µM of inhibitor at different reaction times (0, 2, 20, 60 and 180 min), the reaction was quenched by adding 2 µL of 4% v/v TFA to 10 µL reaction volume and subsequently measured by LC-MS as described above. The observed occupancy percentage over time at a given inhibitor concentration was fit to the following equation using GraphPad Prism:

$$\%\, conversion = \frac{v_i}{k_{obs}}[1 - \exp(-k_{obs} * t)]$$

The obtained k_{obs} values were then plotted as a function of inhibitor concentration and fit to the following equation:

$$k_{obs} = \frac{k_{inact}\,[I]}{[I] + K_I}$$

to yield the k_{inact} and K_I.[26]

4. References

[1] J. M. Ostrem, U. Peters, M. L. Sos, J. A. Wells, K. M. Shokat, *Nature* **2013**, *503*, 548–551.

[2] Schrödinger 2017-3, Schrödinger, LLC, New York, NY, **2017**.

[3] G. M. Sastry, M. Adzhigirey, T. Day, R. Annabhimoju, W. Sherman, *J. Comput.-Aided Mol. Des.* **2013**, *27*, 221–234.

[4] M. P. Jacobson, D. L. Pincus, C. S. Rapp, T. J. Day, B. Honig, D. E. Shaw, R. A. Friesner, *Proteins: Struct., Funct., Bioinf.* **2004**, *55*, 351–367.

[5] K. J. Bowers, D. E. Chow, H. Xu, R. O. Dror, M. P. Eastwood, B. A. Gregersen, J. L. Klepeis, I. Kolossvary, M. A. Moraes, F. D. Sacerdoti, J. K. Salmon, Y. Shan, D. E. Shaw, in *SC '06: Proceedings of the 2006 ACM/IEEE Conference on Supercomputing*, Tampa, Florida, **2006**.

[6] H. Berendsen, J. Postma, W. van Gunsteren, J. Hermans, Reidel, Dordrecht, Jerusalem, Israel, **1981**.

[7] E. Harder, W. Damm, J. Maple, C. Wu, M. Reboul, J. Y. Xiang, L. Wang, D. Lupyan, M. K. Dahlgren, J. L. Knight, J. W. Kaus, D. S. Cerutti, G. Krilov, W. L. Jorgensen, R. Abel, R. A. Friesner, *J. Chem. Theory Comput.* **2016**, *12*, 281–296.

[8] A. Kolb, B. Dünweg, *J. Chem. Phys.* **1999**, *111*, 4453–4459.

[9] S. L. Dixon, A. M. Smondyrev, S. N. Rao, *Chem. Biol. Drug Des.* **2006**, *67*, 370–372.

[10] M. R. Janes, J. Zhang, L.-S. Li, R. Hansen, U. Peters, X. Guo, Y. Chen, A. Babbar, S. J. Firdaus, L. Darjania, J. Feng, J. H. Chen, S. Li, S. Li, Y. O. Long, C. Thach, Y. Liu, A. Zarieh, T. Ely, J. M. Kucharski, L. V. Kessler, T. Wu, K. Yu, Y. Wang, Y. Yao, X. Deng, P. P. Zarrinkar, D. Brehmer, D. Dhanak, M. V. Lorenzi, D. Hu-Lowe, M. P. Patricelli, P. Ren, Y. Liu, *Cell* **2018**, *172*, 578–589.

[11] W. R. Pitt, D. M. Parry, B. G. Perry, C. R. Groom, *J. Med. Chem.* **2009**, *52*, 2952–2963.

[12] a) Glide, release 2017-3, Schrödinger, LLC, New York, NY, **2017**; b) K. Zhu, K. W. Borrelli, J. R. Greenwood, T. Day, R. Abel, R. S. Farid, E. Harder, *J. Chem. Inf. Model.* **2014**, *54*, 1932–1940.

[13] a) J. Wang, T. Hou, X. Xu, *Curr. Comput.-Aided Drug Des.* **2006**, *2*, 287–306; b) W. Wang, O. Donini, C. M. Reyes, P. A. Kollman, *Annu. Rev. Biophys. Biomol. Struct.* **2001**, *30*, 211–243; c) E. Wang, H. Sun, J. Wang, Z. Wang, H. Liu, J. Z. H. Zhang, T. Hou, *Chem. Rev.* **2019**, 119, 9478-9508.

[14] a) FEP+, release 2017-3, Schrödinger, LLC, New York, NY, **2017**; b) Maestro, 3.8.5.19, Schrödinger, LLC, New York, NY, **2017**.

[15] a) M. P. Repasky, M. Shelley, R. A. Friesner, *Curr. Protoc. Bioinf.* **2007**, *8*, 8.12.1–8.12.36; b) R. A. Friesner, J. L. Banks, R. B. Murphy, T. A. Halgren, J. J. Klicic, D. T. Mainz, M. P. Repasky, E. H. Knoll, M. Shelley, J. K. Perry, D. E. Shaw, P. Francis, P. S. Shenkin, *J. Med. Chem.* **2004**, *47*, 1739–1749.

[16] G. J. Martyna, D. J. Tobias, M. L. Klein, *J. Chem. Phys.* **1994**, *101,* 4177-4189.

[17] a) W. G. Hoover, *Phys. Rev. A* **1985**, *31*, 1695-1697; b) S. Nosé, *Mol. Phys.* **1986**, *57* 187-191.

[18] a) A. D. Bochevarov, E. Harder, T. F. Hughes, J. R. Greenwood, D. A. Braden, D. M. Philipp, D. Rinaldo, M. D. Halls, J. Zhang, R. A. Friesner, *Int. J. Quantum Chem.* **2013**, *113*, 2110–2142. b) Force Field Builder, release 2018-2, Schrödinger, LLC, New York, NY.

[19] R. C. Hillig, B. Sautier, J. Schroeder, D. Moosmayer, A. Hilpmann, C. M. Stegmann, N. D. Werbeck, H. Briem, U. Boemer, J. Weiske, V. Badock, J. Mastouri, K. Petersen, G. Siemeister, J. D. Kahmann, D. Wegener, N. Böhnke, K. Eis, K. Graham, L. Wortmann, F. von Nussbaum, B. Bader, *Proc. Natl. Acad. Sci. U. S. A.* **2019**, *116*, 2551–2560.

[20] a) K. M. Sparta, M. Krug, U. Heinemann, U. Mueller, M. S. Weiss, *J. Appl. Crystallogr.* **2016**, *49*, 1085–1092; b) W. Kabsch, *Acta Crystallogr., Sect. D: Biol. Crystallogr.* **2010**, *66*, 125–132.

[21] a) A. J. McCoy, R. W. Grosse-Kunstleve, P. D. Adams, M. D. Winn, L. C. Storoni, R. J. Read, *J. Appl. Crystallogr.* **2007**, *40*, 658–674; b) M. D. Winn, C. C. Ballard, K. D. Cowtan, E. J. Dodson, P. Emsley, P. R. Evans, R. M. Keegan, E. B. Krissinel, A. G. W. Leslie, A. McCoy, S. J. McNicholas,

G. N. Murshudov, N. S. Pannu, E. A. Potterton, H. R. Powell, R. J. Read, A. Vagin, K. S. Wilson, *Acta Crystallogr., Sect. D: Biol. Crystallogr.* **2011**, *67*, 235–242.

[22] G. N. Murshudov, P. Skubak, A. A. Lebedev, N. S. Pannu, R. A. Steiner, R. A. Nicholls, M. D. Winn, F. Long, A. A. Vagin, *Acta Crystallogr., Sect. D: Biol. Crystallogr.* **2011**, *67*, 355–367.

[23] P. Emsley, B. Lohkamp, W. G. Scott, K. Cowtan, *Acta Crystallogr., Sect. D: Biol. Crystallogr.* **2010**, *66*, 486–501.

[24] A. W. Schuttelkopf, D. M. F. van Aalten, *Acta Crystallogr., Sect. D: Biol. Crystallogr.* **2004**, *60*, 1355–1363.

[25] A. A. Lebedev, P. Young, M. N. Isupov, O. V. Moroz, A. A. Vagin, G. N. Murshudov, *Acta Crystallogr., Sect. D: Biol. Crystallogr.* **2012**, *68*, 431–440.

[26] a) J. M. Strelow, *SLAS DISCOV.* **2017**, *22*, 3-20; b) R. Hansen, U. Peters, A. Babbar, Y. Chen, J. Feng, M. R. Janes, L.-S. Li, P. Ren, Y. Liu, P. P. Zarrinkar, *Nat. Struct. Mol. Biol.* **2018**, *25*, 454-462.
